# Glucocorticoids rapidly inhibit cell migration through a novel, non-transcriptional HDAC6 pathway

**DOI:** 10.1242/jcs.242842

**Published:** 2020-06-11

**Authors:** Stephen Kershaw, David J. Morgan, James Boyd, David G. Spiller, Gareth Kitchen, Egor Zindy, Mudassar Iqbal, Magnus Rattray, Christopher M. Sanderson, Andrew Brass, Claus Jorgensen, Tracy Hussell, Laura C. Matthews, David W. Ray

**Affiliations:** 1 Systems Oncology, Cancer Research UK Manchester Institute, Manchester, SK10 4TG, UK; 2 Manchester Collaborative Centre for Inflammation Research, University of Manchester, Manchester, M13 9PT, UK; 3 Lydia Becker Institute of Immunology and Inflammation University of Manchester, Manchester, M13 9PT, UK; 4 Division of Cellular and Molecular Physiology, University of Liverpool, Liverpool, L69 3BX, UK; 5 Platform Sciences, Enabling Technologies, and Infrastructure, University of Manchester, Manchester, M13 9PT, UK; 6 Division of Diabetes, Endocrinology, and Gastroenterology, University of Manchester, Manchester, M13 9PT, UK; 7 Division of Informatics, Imaging, and Data Sciences, Faculty of Biology, Medicine, and Health, University of Manchester, Manchester, M13 9PT, UK; 8 Leeds Institute of Cancer and Pathology, Faculty of Medicine and Health, University of Leeds, Leeds, LS2 9JT, UK; 9 Oxford Centre for Diabetes, Endocrinology and Metabolism (OCDEM), University of Oxford, OX3 7LE, and NIHR Oxford Biomedical Research Centre, John Radcliffe Hospital, Oxford, OX3 9DU, UK

**Keywords:** HDAC6, Cell migration, Glucocorticoid, Microtubule, Quantitative imaging

## Abstract

Glucocorticoids (GCs) act through the glucocorticoid receptor (GR, also known as NR3C1) to regulate immunity, energy metabolism and tissue repair. Upon ligand binding, activated GR mediates cellular effects by regulating gene expression, but some GR effects can occur rapidly without new transcription. Here, we show that GCs rapidly inhibit cell migration, in response to both GR agonist and antagonist ligand binding. The inhibitory effect on migration is prevented by GR knockdown with siRNA, confirming GR specificity, but not by actinomycin D treatment, suggesting a non-transcriptional mechanism. We identified a rapid onset increase in microtubule polymerisation following GC treatment, identifying cytoskeletal stabilisation as the likely mechanism of action. HDAC6 overexpression, but not knockdown of αTAT1, rescued the GC effect, implicating HDAC6 as the GR effector. Consistent with this hypothesis, ligand-dependent cytoplasmic interaction between GR and HDAC6 was demonstrated by quantitative imaging. Taken together, we propose that activated GR inhibits HDAC6 function, and thereby increases the stability of the microtubule network to reduce cell motility. We therefore report a novel, non-transcriptional mechanism whereby GCs impair cell motility through inhibition of HDAC6 and rapid reorganization of the cell architecture.

This article has an associated First Person interview with the first author of the paper.

## Abstract (precis)

**Summary:** A novel inhibitory mechanism of glucocorticoid (GC) action on cell migration is dependent on the α-tubulin deacetylase HDAC6, which has implications for the design of novel GCs with improved therapeutic benefit.

## INTRODUCTION

Glucocorticoids (GCs) are steroid hormones that regulate a range of biological functions essential for life, including normal homeostasis, glucose metabolism, resolution of inflammation and development (McMaster et al., 2008; Tu et al., 2018; Tanaka et al., 2017). GCs exert their biological effects through the ubiquitously expressed glucocorticoid receptor (GR; also known as NR3C1), a ligand-inducible transcription factor of the nuclear hormone receptor superfamily (Hollenberg et al., 1985). Synthetic GCs (including dexamethasone, fluticasone furoate and prednisolone) are powerful anti-inflammatory and immunosuppressive drugs that are widely prescribed in the clinic to treat a variety of ailments (Donn et al., 2007; Smoak and Cidlowski, 2004; Xing et al., 2015). However, the pleiotropic action of GCs leads to severe off-target effects that severely limits prolonged clinical use, including osteoporosis, diabetes and impaired wound healing (Zhou and Cidlowski, 2005; Abell et al., 2015). For this study, we investigated the mechanism underlying GC impairment of wound healing and by extension the inhibition of cell migration, which is implicated in impaired wound healing (Matsubayashi et al., 2004).

GCs are known to inhibit the migration of various cell types, yet with an unrecognised mechanism of action (Fietz et al., 2017; Murakami et al., 1998). Regulation of cell motility has often been attributed to reorganization and stabilisation of the actin and microtubule networks (Akhshi et al., 2014; Yumura et al., 2013; George et al., 2013; DeFea, 2013; Yang et al., 2010). The actin network generates the propulsive force necessary for front-end protrusion and rear-end retraction of cells, facilitating cell movement (Kaverina and Straube, 2011; Ridley et al., 2003). The actin and microtubule networks can cross-talk, which impacts persistent cell movement through myosin convergence and focal adhesion turnover (Wu and Bezanilla, 2018; Schneider and Persson, 2015; Juanes et al., 2017). Cell movement is highly dependent on the state of microtubule dynamic stability (Pitaval et al., 2017). Microtubule stability is regulated by acetylation of lysine-40 (K40) on α-tubulin, with acetylated α-tubulin being most abundant in stable microtubules (Piperno et al., 1987; Zhang et al., 2003). Deacetylation of α-tubulin is catalysed by histone deacetylase-6 (HDAC6) and modulation of HDAC6 activity impacts cell migration by altering the dynamics of the microtubule network (Hubbert et al., 2002; Zhang et al., 2007; Shi et al., 2015). Overexpression of HDAC6 increases cell motility by regulating microtubule-dependent migration (Ridley et al., 2003; Wu and Bezanilla, 2018). GRs are known to be bound to the cytoskeleton, which is important to permit rapid ligand-induced nuclear translocation of the activated GR, among other functions (Mayanagi et al., 2008; Hong et al., 2011; Fitzsimons et al., 2008; Dvorak et al., 2004; Akner et al., 1995). In addition, HDAC6 deacetylates heat-shock protein-90 (Hsp90), which is vital for GR maturation and maintaining the receptor in a ligand-binding state (Tao et al., 2018; Ai et al., 2009; Kovacs et al., 2005; Rajapandi et al., 2000). GR is also reported to physically associate with HDAC6 in the nucleus (Govindan, 2010; Rimando et al., 2016). We hypothesised that GCs inhibit cell migration by altering the stability of the microtubule network via HDAC6, likely through an inhibitory interaction facilitated by the interconnected substrate Hsp90.

We now show that GCs act rapidly, and in a non-transcriptional mechanism, to inhibit cell migration. Furthermore, GCs impair HDAC6 regulation of the microtubule network to increase the proportion of short-steps and reduce the proportion of long-steps; modelled as a change in α-stable distribution parameters. There was evidence that activated GR impacted the movement of HDAC6 in target cells, and for cross-coupling of the GR and HDAC6, as shown with fluorescence cross-correlation spectroscopy (FCCS) but not co-immunoprecipitation, suggesting a highly dynamic and unstable interaction between a small proportion of the intracellular HDAC6 pool and the activated GR.

## RESULTS

### GR agonists and antagonists inhibit cell migration

**Fig. 1.**
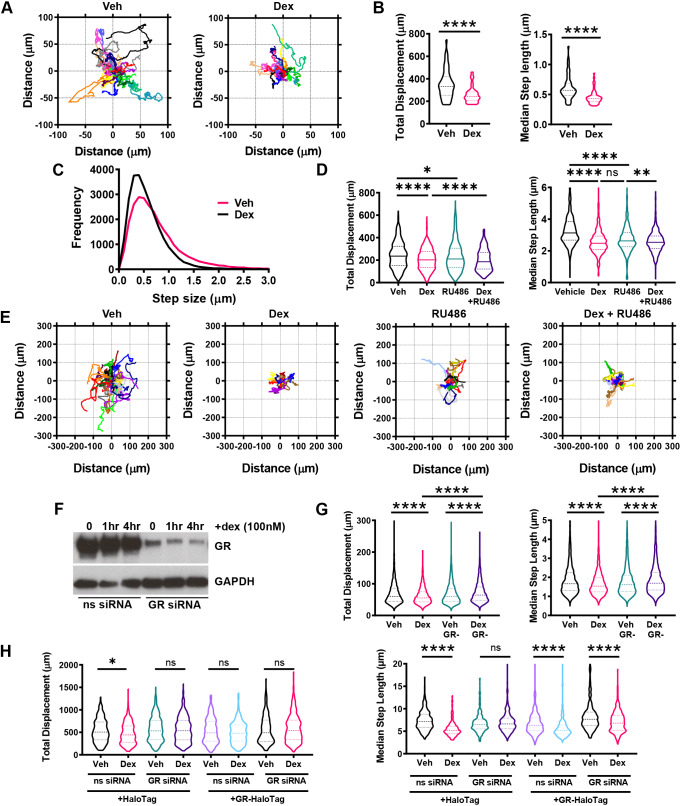
**GR agonists and antagonists inhibit cell migration.** (A–C) Cell migration data for A549 cells that were transiently transfected with 0.5 µg pBOS-H2B-GFP and incubated for 24 h at 37°C/5% CO_2_ prior to imaging. Cells were treated with vehicle (Veh; DMSO) and dexamethasone (dex; 100 nM) and images acquired every 5 min for 48 h. Data displayed represents the first 24 h of tracking. (A) Rose plots of A549 cell displacement (µm) in response to 24 h of vehicle and dex (100 nM) treatment. Each coloured line represents the displacement of one cell from its point of origin. Each rose plot is representative of 20 randomly chosen A549 cells. (B) Violin plots of total displacement (µm; the overall distance moved by every cell tracked) and median step length (µm; the median of all the distances move by a cell between each image acquisition over the entire duration of tracking) for A549 cells in response to 24 h of vehicle and dex (100 nM) treatment. Migration data is representative of 52 individual cells for vehicle treatment and 54 individual cells for dex treatment, across two independent experiments. Dashed lines represent the median±interquartile range (IQR) (unpaired *t*-test; ****P<0.0001). (C) Frequency distribution curves of all A549 cell step lengths (µm) in response to 24 h of vehicle and dex (100 nM) treatment. (D,E) Cell migration data for A549 cells that were tracked using brightfield microscopy. Cells were treated with vehicle (DMSO) and dex (100 nM) and images acquired every 10 min for 24 h, with data displaying all 24 h of tracking. (D) Violin plots showing the total displacement (µm) and median step length (µm) of A549 cells in response to 24 h of vehicle, dex (100 nM), RU486 (100 nM), and dex+RU486 (both 100 nM) co-treatment. Migration data is displayed as the median±IQD and represents all cells analysed over three independent experiments (Kruskal–Wallis non-parametric test followed by Dunn's multiple comparisons test; *P=0.0342, **P=0.0076, ****P<0.0001). (E) Rose plots of A549 cell displacement (µm) in response to 24 h of dex (100 nM), RU486 (100 nM), RU486 (100 nM), and a dex+RU486 (both 100 nM) co-treatment. Each coloured line represents the displacement of one cell from its point of origin. Each rose plot is representative of 20 randomly chosen A549 cells. (F) Western blot of GR and GAPDH protein expression in control (non-silencing siRNA-treated) and GR knockdown (GR siRNA#6-treated) A549 cells in response to 1 and 4 h of vehicle and dex (100 nM) treatment. siRNA treatments were performed over 48 h. Western blot image is representative of two independent experiments. (G) Cell migration data for A549 cells that were tracked using brightfield microscopy. Cells were transiently transfected with siRNA targeting GR or a non-targeting siRNA negative control for 48 h. GR-knockdown cells were then treated with vehicle (DMSO) or dex (100 nM) and images acquired every 10 min for 6 h, with data displaying all 6 h of tracking. Violin plots of total displacement (µm) and median step length (µm) of control and GR knockdown A549 cells in response to 6 h of vehicle and dex (100 nM) treatment. Migration data shown as median±IQD and represents all cells analysed over three independent experiments (Kruskal–Wallis non-parametric test followed by Dunn's multiple comparisons test; ****P<0.0001). (H) Cell migration data for A549 cells that were tracked using fluorescence microscopy based on Rhodamine expression. GR-knockdown cells were transiently transfected with HaloTag empty vector (2 µg) or HaloTag–GR (2 µg) for 24 h and then treated with vehicle (DMSO) or dex (100 nM) for 24 h. Cells were labelled overnight with HaloTMRDirect ligand (100 nM) that labels HaloTag proteins with the Rhodamine fluorophore. Images were acquired every 10 min, with data displaying 24 h of tracking. Violin plots of total displacement (µm) and median step length (µm) of control and GR knockdown A549 cells overexpressing 2 µg empty-pHaloTag control or 2 µg HaloTag-GR in response to 24 h of vehicle or dex (100 nM) treatment. GR knockdown was siRNA-mediated over 48 h, alongside a non-silencing siRNA negative control. Migration data as the median±IQD and represents all cells analysed over three independent experiments (Kruskal–Wallis non-parametric test followed by Dunn's multiple comparisons test; *P=0.0159, ****P<0.0001). ns, not significant.

Dexamethasone (dex), a synthetic GR agonist, potently inhibits the migration of A549 cells tracked by immunofluorescence imaging of GFP-labelled histone 2B (H2B) (Fig. 1A), causing a marked reduction in total displacement (median=141.0 µm) and step length (median=0.43 µm) compared to vehicle-treated controls (median total displacement=165.8 µm; median step length=0.52 µm) (Fig. 1B). There was a significant increase in small step size and corresponding decrease in large step size typical of inhibited cell migration, revealing a shift in the cell walk properties (Fig. 1C). Dex also inhibited A549 cell migration at the population level in wound healing and chemotaxis assays performed over 48 h (Fig. S1A,B).

In separate experiments tracking cells using brightfield microscopy, cell migration was also significantly inhibited upon treatment with the GR antagonist RU486 (vehicle-treated control median total displacement=235.5 µm; median step length=2.68 µm compared to RU486 median total displacement=210.3 µm; median step length=2.63 µm) (Fig. 1D; Fig. S1C), which was surprising, and indeed RU486 did not antagonise the inhibitory effect of dex (Fig. 1D,E). RU486 is a competitive GR antagonist that binds and induces GR nuclear translocation, but then recruits corepressors, including NCoRs, to block transcription (Fig. S1D). In A549 cells, we did not detect statistically significant GR transactivation with RU486 treatment (Fig. S1D).

The overlapping actions of dex and RU486 on cell migration suggest a common mechanism of action, but one that requires the GR, and not the transcriptional regulatory actions of the GR. As the effects seen were so unexpected, we also tested the requirement of the GR, using siRNA (Fig. 1F; Fig. S1E), which confirmed the need for GR (Fig. 1G), a conclusion strengthened by complementation assays using siRNA-resistant HaloTag-GR (Fig. 1H; Fig. S1E,F). In addition, scratch wound assays of A549 cells demonstrated the inhibitory effect of the endogenous GC hydrocortisone in addition to dex and RU486 on cell migration, suggesting a ligand-dependent mechanism of action (Fig. S1C).

GCs also inhibit the migration of many other cell types. To test the broader applicability of our findings we used primary peritoneal macrophages from GR^f/f^ and LysM-creGR^f/f^ mice (Fig. S1F). The cells expressing wild-type GR show inhibition of migration upon GC stimulation, but this inhibitory effect is completely lost in the GR-null cells.

### The α-stable distribution models A549 motion

The distribution of step lengths in vehicle- and dex-treated conditions (Fig. 1C) showed the distinctive walk pattern indicative of an α-stable distribution, characterised by four parameters that describe the stability exponent (α), skewness (β), scale (γ) and location (δ) (Salas-Gonzalez et al., 2013; Burnecki et al., 2012). GC treatment reduced median step length, signified by a left-shift in the frequency distribution curve (measured by a reduction in δ parameter). α-Stable parameters were derived using MATLAB, showing that A549 cell movement adopted an α-stable distribution irrespective of GC treatment (Fig. S2A,B). These changes in parameters show that the movement of vehicle-treated A549 cells primarily consists of small steps occasionally interspersed with larger relocating or searching steps. GC alters these parameters inhibiting the low frequency, large displacement searching movements.

### Synthetic selective GR ligands exhibit similar effects to conventional GC

**Fig. 2.**
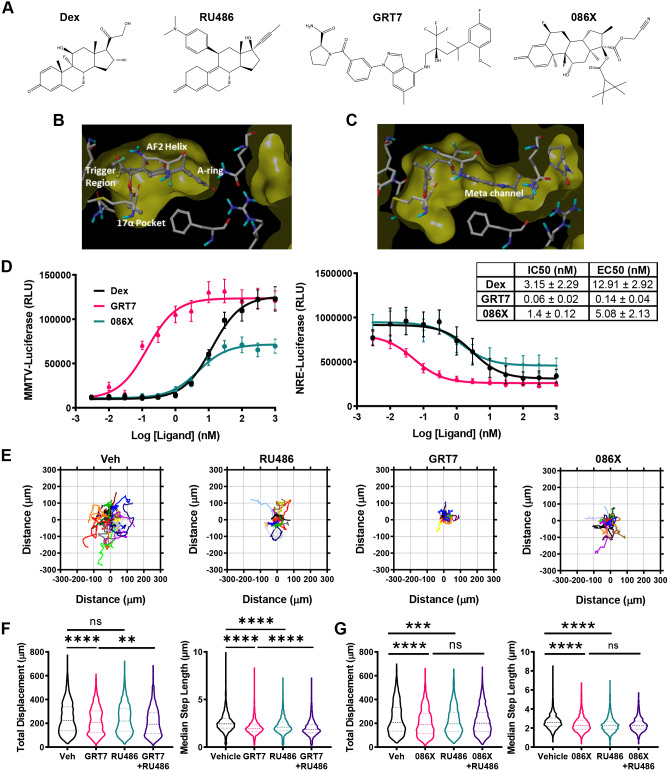
**Selective glucocorticoids also inhibit cell migration.** (A) 3D chemical structures of dex, RU486, GRT7 and GW870086X (086X). (B) Crystal structures of the GR ligand-binding domain (LBD) bound to dex with trigger region annotated. (C) Crystal structure of the GR LBD bound to GRT7 annotated with regions altered by ligand-binding (meta channel). (D) Luciferase activity of the MMTV-luciferase or NRE-luciferase reporter genes which were transiently transfected into A549 cells over 24 h. Luciferase activity was monitored after 24 h treatment with vehicle, dex (red), GRT7 (brown), and 086X (blue). Data (RLU) are shown as mean±s.e.m. (*n*=3). Ligand potencies are displayed as mean±s.d. IC_50_ and EC_50_ values. (E,F) Cell migration data for A549 cells that were tracked using brightfield microscopy. Cells were treated with vehicle (Veh; DMSO), GRT7 (100 nM), GW870086X (100 nM) and RU486 (100 nM), and images acquired every 10 min for 24 h, with data displaying all 24 h of tracking. (E) Rose plots of A549 cell displacement (µm) in response to 24 h potency-matched vehicle, RU486 (100 nM), GRT7 (3 nM) and 086X (100 nM). Each coloured line represents the displacement of one cell from its point of origin. Each rose plot is representative of 20 randomly chosen A549 cells. (F) Violin plots of total displacement (µm) and median step length (µm) of A549 cells in response to 24 h of vehicle, 086X (100 nM), RU486 (100 nM), and 086X+RU486 (both 100 nM) co-treatment. Migration data shown as median±interquartile range (IQR; dashed lines) and represents all cells analysed over three independent experiments (Kruskal–Wallis non-parametric test followed by Dunn's multiple comparisons test; **P=0.0015, ****P<0.0001). (G) Violin plots of total displacement (µm) and median step length (µm) of A549 cells in response to 24 h of vehicle, GRT7 (3 nM), RU486 (100 nM), and GRT7+RU486 (both 100 nM) co-treatment. Migration data shown as median±IQR and represents all cells analysed over three independent experiments (Kruskal–Wallis non-parametric test followed by Dunn's multiple comparisons test; ***P=0.0001, ****P<0.0001). ns, not significant.

In view of the inhibitory effect of RU486 on cell migration, the study was extended to further non-steroidal GR ligands with unique pharmacological properties (Trebble et al., 2013; Schiller et al., 2014), and we selected a panel based on high affinity and specificity (Fig. 2A). For example, GRT7 extends into the meta channel of the GR ligand-binding domain (LBD) driving slower kinetics of activation, but more-potent transcriptional induction (Fig. 2B–D) (Trebble et al., 2013). GW870086X (086X), is a selective GR modulator (SeGRM), deficient in transactivation function (Fig. 2D). All the GR ligands tested similarly reduced A549 cell displacement (Fig. 2E), affecting both total displacement (086X=188.5 µm; vehicle=208.3 µm) (GRT7=208.3 µm; vehicle=224.1 µm) and median step length (086X=2.28 µm; vehicle=2.58 µm) (GRT7=1.95 µm; vehicle=2.44 µm). Cell walk properties were similarly inhibited (Fig. 2F,G; Figs S2C–E, S3E,F). As with dex, RU486 did not antagonise the inhibition of cell migration with GRT7 or O86X (GRT7+RU486 median total displacement=192.6 µm; GRT7+RU486 median step length=1.86 µm; 086X+RU486 median total displacement=201.3 µm; 086X+RU486 median step length=2.26 µm). In the study with GRT7, we did see a slight potentiation of the inhibitory effect when RU486 was added to the GRT7 compound, but in this series of studies the RU486 effect was slightly less than seen in other repeats when we analysed total cell displacement. However, the reduction in median step length was striking, and consistent throughout. Additional analysis was conducted using the high potency, steroidal GR agonist fluticasone propionate (FP), with similar effects on movement (Fig. S3A–C) and α-stable distribution parameter changes confirming altered walk properties (Fig. S3D–F).

### GC inhibits migration independently of gene transcription

**Fig. 3.**
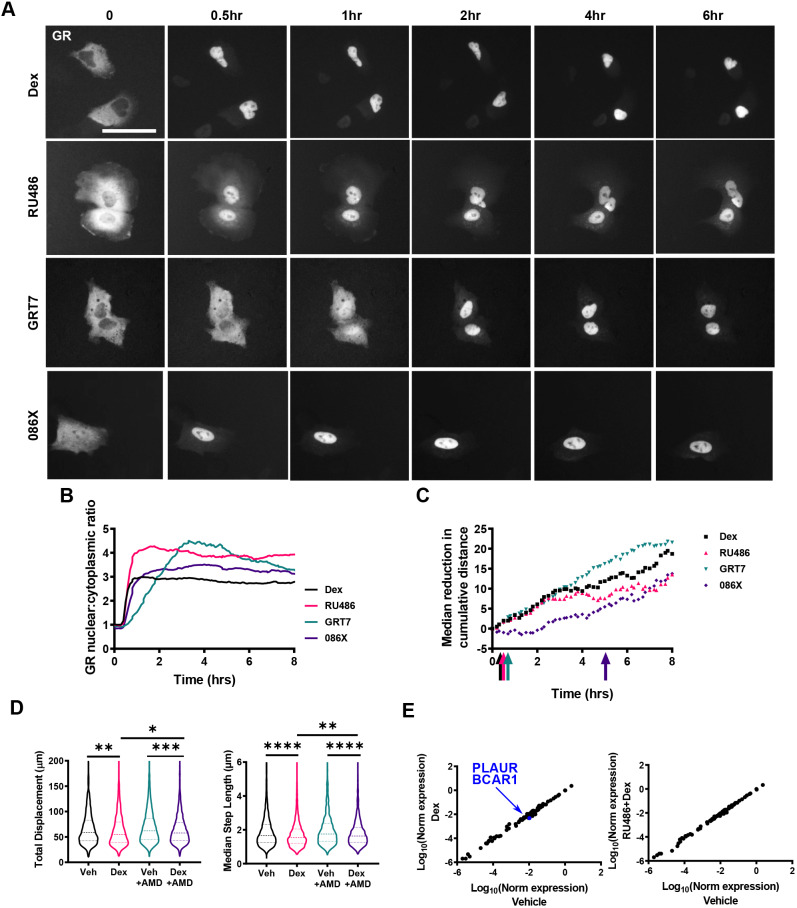
**Ligand-specific regulation of migration kinetics.** (A) Representative widefield images of HaloTag–GR nuclear accumulation in A549 cells over 6 h of dex (100 nM), RU486 (100 nM), GRT7 (3 nM), and 086X (100 nM) treatment. A549 cells were transiently transfected with 250 ng HaloTag–GR and labelled with Halo^®^TMRDirect™ ligand (100 nM). Images are representative of three independent experiments. (B) Nuclear:cytoplasmic ratio of GR localisation in response to potency-matched treatment of dex (100 nM), RU486 (100 nM), GRT7 (3 nM) and 086X (100 nM) over 8 h. GR localisation was quantified using ImageJ. (C) Non-parametric rank-sum test of A549 cell migration (cumulative distance) signifying the earliest timepoint (coloured arrows) at which migration is statistically different [*P*<0.05; Mann–Whitney U (Wilcoxon rank sum) test] in response to dex (100 nM), RU486 (100 nM), GRT7 (3 nM), and 086X (100 nM) compared to vehicle-treated controls. (D,E) Cell migration data for A549 cells that were tracked using brightfield microscopy. Cells were pre-treated for 1 h with vehicle (DMSO) or actinomycin D (1 µg/ml) before treating with vehicle (DMSO) or dex (100 nM), and images were acquired every 10 min for 4 h, with data displaying all 4 h of tracking. (D) Violin plots of total displacement (µm) and median step length (µm) of A549 cells in response to 1 h pre-treatment with vehicle or actinomycin D (1 µg/ml) and subsequent 4 h treatment with vehicle or dex (100 nM). Migration data shown as the median±interquartile range (IQR; dashed lines) and represents all cells analysed over two independent experiments (Kruskal–Wallis non-parametric test followed by Dunn's multiple comparisons test; *P=0.01, **P<0.004, ***P=0.0001, ****P<0.0001). (E) RT^2^ qPCR array of genes that regulate cell migration in response to 4 h of dex (100 nM), and a dex plus RU486 (both 100 nM) co-treatment. Each data point represents an individual gene. Values on the scatter plot represent log_10_ (normalised expression). Genes in black have a fold change over the vehicle control less than 2 and greater than 0.5. Genes with a fold change less than 0.5 (downregulated) are indicated in blue.

Through dynamic cell tracking (Fig. S1C), we noted that the GC effect had a rapid onset, an observation not identified previously using fixed end-point assays (Fig. S1A,B). When we analysed displacement at earlier time points there was a significant reduction by 12 h, and a trend was seen even by 4 h, although this did not reach significance (Fig. S4A–C). To investigate the time course of the GC response in greater detail, we used a non-parametric rank-sum test to determine the earliest time-point at which cell migration was significantly reduced following treatment with each GC and compared this to the dynamics of ligand-induced GR nuclear translocation (Fig. 3A–C). Dex and RU486 both inhibited migration within 60 min of administration; and GRT7, which does not lead to translocation of the GR until 3 h post treatment, also inhibits migration by 60 min. O86X, which induces the most rapid GR translocation, only inhibits migration after 5 h (indicated by the coloured arrows, Fig. 3C). The kinetics of nuclear translocation were inverse to those for migration inhibition. The rapid onset of action, with response preceding nuclear translocation, as in the case of the GRT7 ligand, suggested a non-conventional mechanism of action, such as a cytoplasmic, non-genomic circuit. A non-conventional mechanism of action is also supported by the lack of antagonism seen with the use of RU486 in the presence of the agonists, despite the absolute requirement for the presence of the GR.

In order to test the importance of new gene transcription for the altered cell migration we used actinomycin D pre-treatment. A 1-h treatment with actinomycin D was enough to block the transcriptional activation function of the GR (Fig. S5A), and these studies again confirmed the complete lack of agonist activity seen with RU486 in these cells, under these conditions. Under these treatment conditions, we were able to see the same change in cell migration with a 4-h incubation with dex both with and without actinomycin D blockade of gene transcription (Fig. 3D). Actinomycin D did not affect cell migration independently.

We also profiled changes in gene expression of a panel of genes known to control cell migration. In this study, we again selected the 4-h time point as the most discriminating, as the change in cell migration by this point does not require a change in gene expression. Here, we observed only two genes (*PLAUR* and *BCAR1*) to be downregulated by a 4 h incubation with dex. This repression was opposed by RU486, as expected for a conventional GR antagonist. As GR agonist and antagonist regulated these two genes in opposite directions, this implies that they are not relevant to the migration phenotype we are observing, as we show that no new gene transcription is required for the effect, and also that the migration phenotype is both observed with RU486 treatment, and also that RU486 treatment does not oppose the actions of agonists, such as dex, when the cell migration phenotype is observed (Fig. 3E).

### GC treatment rapidly stabilises microtubules

**Fig. 4.**
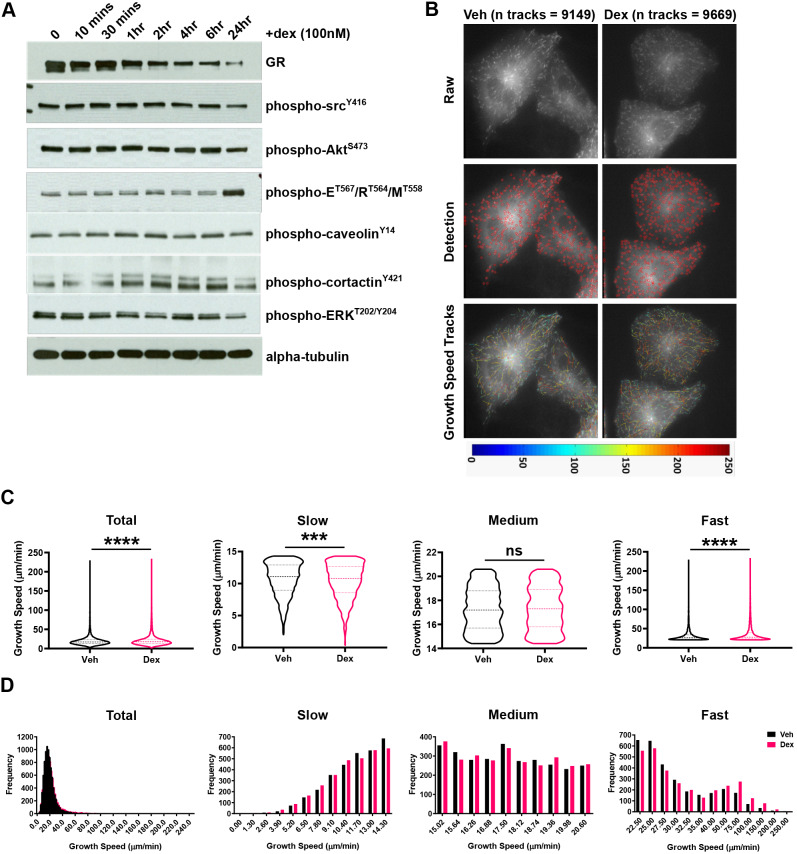
**Glucocorticoids stabilise the microtubule network.** (A) Western blot of total GR, phospho-Src^Y416^, phospho-Akt^S473^, phospho-Ezrin^T567^/Radixin^T564^/Moesin^T558^ (E/R/M), phospho-caveolin^Y14^, phospho-cortactin^Y421^, phospho-ERK^T202/Y204^ and total α-tubulin protein expression in response to dex (100 nM) as a time series (0, 10 min, 30 min, 1 h, 2 h, 4 h, 6 h, and 24 h). Western blots are representative of three independent experiments. (B) Representative images of A549 cells transiently transfected with EB3–GFP over 24 h and then treated with vehicle (Veh; DMSO) and dex (100 nM) for 4 h. Cells were selected at random based on expression of EB3–GFP and then microtubule comets (microtubule plus-ends expressing EB3–GFP) were imaged every 0.5 s for 60 s (total 120 images acquired; top panel). Microtubule dynamics were determined from the 60 s EB3–GFP time series using plusTipTracker software (MATLAB; middle and bottom panels). Fluorescently labelled microtubule plus-ends were first detected (middle panel) and then microtubule growth events were tracked (bottom panel), and dynamics determined. Tracks are represented by a colour-coded heat map (indicating growth speed μm/min) imposed upon the raw image (bottom panel). (C) Violin plots of microtubule growth speed (µm/min) in A549 cells following a 4 h vehicle and dex (100 nM) treatment. Growth speed data is shown entirely (upper left panel; total) or split into tertiles according to speed (designated slow, medium or fast). Each bin size corresponds to one-third of all growth speed events following vehicle treatment, and was applied to the dex-treated data set. Data is representative of three independent experiments (*n*=3); 9149 (vehicle-treated) and 9669 (dex-treated) growth events, from 10 cells per condition were tracked and analysed. Results are median±interquartile range (dashed lines; Mann–Whitney non-parametric test; ***P=0.0002, ****P<0.0001; ns, not significant). (D) Histograms displaying the frequency of microtubule growth speeds (µm/min) in A549 cells following a 4 h vehicle and dex (100 nM) treatment. Growth speed data is shown entirely (upper left panel; total) or split into tertiles as described in C. Data for vehicle-treated cells is indicated by red bars and for dex-treated cells by blue bars.

To investigate the mechanism explaining the early onset, non-transcriptional response of cell migration to GC treatment, we profiled the activation status of a panel of candidate proteins (Fig. 4A). We observed a reduction in phosphorylated ERK1/2 (phospho-ERK; ERK1 is also known as MAPK3 and ERK2 as MAPK1), and an induction in phosphorylated ezrin, radixin and moesin (phospho-ERM), but these changes were not seen until 24 h post GC stimulation. This suggests that these molecules are not involved in mediating the very rapid responses we see in response to GC treatment, and their change in status may follow on from rather than drive the change in cell phenotype. To analyse rapid responses, we turned to live-cell imaging of the cytoskeleton, to determine whether changes in cytoskeletal architecture could be observed.

To investigate the actin and MT cytoskeletal networks we examined the effect of dex on MT dynamics by following an GFP-tagged MT plus-end (+TIP) binding protein, EB3 (also known as MAPRE3) (Fig. 4B). GC drives an increase in overall MT growth speed (vehicle median=17.2 μm/min; dex median=17.7 μm/min), consistent with stabilisation of microtubules (Fig. 4C, total). To analyse the impact of GCs on MT dynamics in more depth, the growth speed data was subdivided into thirds, designated slow, medium and fast (Fig. 4C,D). Dex resulted in an increased median speed (vehicle=26.2 μm/min; dex=27.6 μm/min) in the fast speed events, with a decreased speed (vehicle=11.1 μm/min, dex=10.8 μm/min) observed in the slow speed events, while no significant difference was detected at medium speed (Fig. 4C). This indicates a shift to more rapidly polymerising microtubules in the presence of dex. A concomitant increase in the frequency of fast growth speed events was also detected (Fig. 4D, 40 μm/min onwards). We attempted direct measurement of acetylation of α-tubulin, a marker of microtubule (MT) stability. We did see small increases at early time points (<10 min), but the effect size was small, and on further investigation the effect was not robust in replication. Therefore, we do not have convincing evidence that tubulin acetylation changes in response to GC, but the changes in microtubule growth were very robust and highly significant.

### GC alter microtubule dynamics by inhibiting HDAC6

**Fig. 5.**
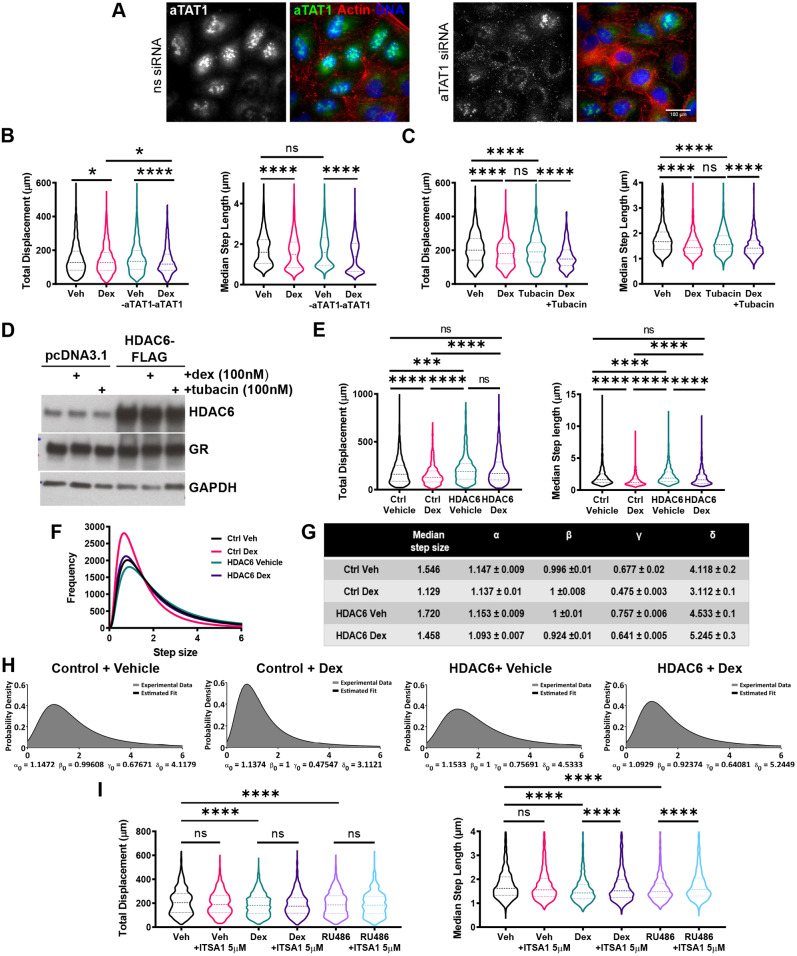
**GC inhibits HDAC6 to regulate cell migration.** (A) Representative images of control and αTAT1-knockdown A549 cells stained via immunofluorescence for αTAT1 (green) and F-actin (red). Nuclei were stained with DAPI (blue). αTAT1-knockdown was siRNA mediated over 48 h, alongside a non-silencing siRNA (ns siRNA) negative control. Images are representative of three independent experiments. Widefield images were acquired on a Delta Vision RT (Applied Precision, GE Healthcare) restoration microscope at 20× magnification. (B) Cell migration data for A549 cells that were tracked using brightfield microscopy. Cells were transiently transfected with siRNA targeting αTAT1 or a non-targeting siRNA negative control for 48 h. Images were acquired every 10 min, with data displaying all 24 h of tracking. Violin plots of total displacement (µm) and median step length (µm) of control and αTAT1 knockdown A549 cells in response to 24 h of vehicle and dex (100 nM) treatment. Migration data shown as median±interquartile range (IQR; dashed lines) and represents all cells analysed over two independent experiments (Kruskal–Wallis non-parametric test followed by Dunn's multiple comparisons test; *P<0.003; ****P<0.0001). (C) Cell migration data for A549 cells that were tracked using brightfield microscopy. Images were acquired every 10 min, with data displaying all 24 h of tracking. Violin plots of total displacement (µm) and median step length (µm) of A549 cells in response to 24 h of vehicle, dex (100 nM), tubacin (100 nM), and dex plus tubacin (both 100 nM) co-treatment. Migration data shown as median±IQR and represents all cells analysed over two independent experiments (Kruskal–Wallis non-parametric test followed by Dunn's multiple comparisons test; ****P<0.0001). (D) Western blot of HDAC6 and GR protein expression in A549 cells transiently transfected with pcDNA3.1 (empty vector control) or HDAC6–FLAG and treated with 1 h of vehicle, dex (100 nM) or tubacin (100 nM). Western blot is representative of two independent experiments. (E) Cell migration data for A549 cells that were tracked using fluorescence microscopy based on GFP expression. Cells were transiently co-transfected with H2B–GFP (0.25 µg) and HDAC6–FLAG (0.25 µg) for 24 h. Cells were then treated with vehicle (DMSO) or dex (100 nM), and images were acquired every 10 min for 24 h, with data displaying 24 h of tracking. Violin plots of total displacement (µm) and median step length (µm) of control (H2B–GFP) and HDAC6 overexpressing (H2B–GFP+HDAC6–FLAG) A549 cells in response to 24 h of vehicle and dex (100 nM) treatment. Migration data shown as median±IQR and represents all cells analysed over two independent experiments (Kruskal–Wallis non-parametric test followed by Dunn's multiple comparisons test; ***P=0.0004, ****P<0.0001). (F) Frequency distribution curves of all A549 cell step lengths (µm) in cells overexpressing empty control vector or HDAC6, in response to vehicle and dex (100 nM) treatment. (G) Estimated mean±s.d. α-stable distribution parameters of A549 cells overexpressing empty control vector or HDAC6 in response to vehicle and dex (100 nM) treatment. α-Stable parameters were derived by analysing experimentally determined step length data in MATLAB. Parameters estimated were for media step length (µm), stability exponent (α), skewness (β), scale (γ) and location (δ). Standard deviation estimates were generated by parameterising 100 randomly sampled subsets of the 15,000 values from the original data sets. (H) Probability density function (PDF) plots of experimental step length data (black line) and PDF plots generated from estimated α-stable distribution parameters (dark grey) in response to vehicle and dex (100 nM) treatment. Plots generated in MATLAB. (I) Cell migration data for A549 cells that were tracked using fluorescence microscopy based on GFP expression. Cells were treated with vehicle (DMSO) or ITSA1 (5 µM) in combination with vehicle (DMSO), dex (100 nM) or RU486 (100 nM) for 24 h. Images were acquired every 10 min for 24 h, with data displaying 24 h of tracking. Violin plots of total displacement (µm) and median step length (µm) of A549 cells in response to 24 h vehicle, dex (100 nM), RU486 (100 nM), ITSA1 (5 µM)+vehicle co-treatment, ITSA1 (5 µM)+dex (100 nM) co-treatment, and ITSA1 (5 µM)+RU486 (100 nM) co-treatment. Migration data shown as median±IQR and represents all cells analysed over two independent experiments (Kruskal–Wallis non-parametric test followed by Dunn's multiple comparisons test; ****P<0.0001). ns, not significant.

Acetylation of tubulin is tightly controlled by the α-tubulin acetyltransferase αTAT1 and the tubulin deacetylase HDAC6, making these two enzymes candidate effectors. We examined αTAT1-knockdown cells (Fig. 5A), but found no effect, suggesting an alternative mechanism of GR action (Fig. 5B). Tubacin, a selective HDAC6 inhibitor, not only mimicked the inhibitory effect of dex (Fig. 5C), but also showed no additive effect in co-treatment protocols, suggesting a convergent mechanism of action. Therefore, we analysed the effect of augmenting HDAC6 expression (Fig. 5D), which increased both the displacement and median step length of cells and rendered cells resistant to GC (Fig. 5E,F). The α-stable distribution parameters changed in response to HDAC6 overexpression with cells adopting a higher proportion of large walk steps indicating increased cell migration relative to the controls, which was unchanged following administration of dex (Fig. 5G,H). The pan-HDAC activator ITSA1 (Haggarty et al., 2003) also reversed the GC migration phenotype, confirming the contributory role of HDAC6 in this mechanism (Fig. 5I).

### GR and HDAC6 are complexed together in the cytoplasm

There was no evidence of altered HDAC6 subcellular trafficking in response to dex with the enzyme remaining predominantly cytoplasmic (Fig. S6A). Co-immunoprecipitation studies also failed to identify GR and HDAC6 in complex together (Fig. S6B), despite previous reports of co-binding and interactive function on gene repression in the nucleus (Rimando et al., 2016; Govindan, 2010). However, we did detect a change in HDAC6 interactions with actin components of the cytoskeleton in response to GC (Fig. S6C,D); although these correlative studies do not provide evidence for physical, or functional interaction, they do suggest molecular proximity.

**Fig. 6.**
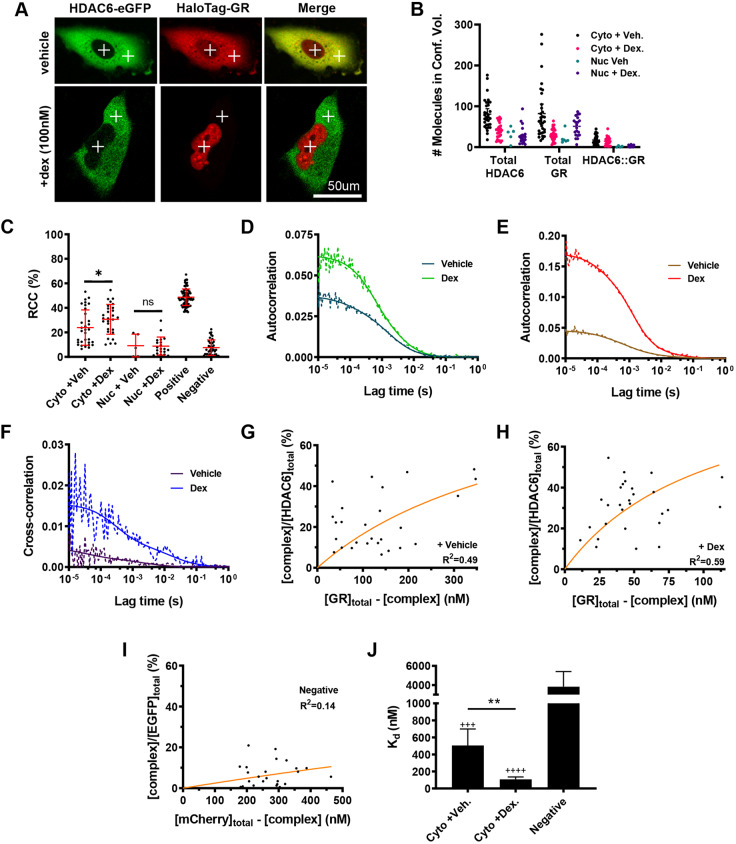
**Activated GR and HDAC6 interact within the cytoplasm.** (A) Confocal images of A549 cells co-transfected with HDAC6–eGFP and HaloTag–GR treated or not with dex (100 nM) for 1 h. Confocal volumes designated for fluorescence cross-correlation spectroscopy (FCCS) measurements are indicated as crosses (cytoplasm and nucleus). (B) Number of GR and HDAC6 molecules in response to vehicle and dex (100 nM) within the cytoplasm and nucleus. (C) Relative cross-correlation (RCC) of the fraction of GR bound to HDAC6 (%) in response to vehicle and dex (100 nM) within the cytoplasm and nucleus. A549 cells expressing a fusion of EGFP and mCherry or separate free EGFP and mCherry were taken as positive and negative controls, respectively. *P=0.038 (matched-pairs one-way ANOVA followed by Sidak's multiple comparison test). (D–F) Example autocorrelation curves of HDAC6 (green) and GR (red) in response to vehicle and dex (100 nM) within the cytoplasm and example cross-correlation curves (blue) of HDAC6–GR interaction in response to vehicle and dex within the cytoplasm. Solid and dotted lines represent raw and fitted correlation curves, respectively. (G–I) Binding kinetics of the HDAC6–GR interaction in response to vehicle and dex (100 nM) within the cytoplasm. The fraction of the HDAC6–GR complex compared to total HDAC6–eGFP expressed was plotted as a function of unbound HaloTag-GR and the equation ([complex]/[green]_total_)=([red]_total_−[complex])/(K_d_+[red]_total_−[complex]) fit to determine the *in vivo K*_d_ (orange line). (J) *In vivo K*_d_ of HDAC6–GR interaction in response to vehicle and dex (100 nM) within the cytoplasm. *K*_d_ displayed as mean with 95% c.i. (for comparison of c.i. see Materials and Methods; ^+++^*P*<0.001; ^++++^*P*<0.0001 indicates significant difference from negative control; **P<0.01). FCCS data represent quantification of three independent experiments from >30 cells.

To study the GR–HDAC6 interaction in further detail, we employed real-time fluorescence cross-correlation spectroscopy (FCCS), which is able to discriminate between cellular compartments (marked by crosses, Fig. 6A). We identify a cytoplasmic but not nuclear interaction between GR and HDAC6 as compared to an empty fluorophore negative control (Fig. 6B–F). An approximate 5-fold increase in interaction strength post dex treatment after GR ligand activation was estimated through determination of *in vivo* dissociation constant (*K*_d_) values (Fig. 6G–J).

## DISCUSSION

Although therapeutic GCs are widely used, their diverse actions limit long-term safety. Multiple candidate mechanisms of action have been advanced, with the major focus on how the same activated receptor can both repress and activate different genes in a cell-type-specific context. Gene repression has been a focus of study, as this pathway appears to mediate the beneficial anti-inflammatory and immune suppressive actions of GCs (Ramamoorthy and Cidlowski, 2016). To this end, new partial agonist GR ligands have been developed and tested in the clinic. Such selective GR modulators (SeGRMs) differentiate GR function mainly by affecting the ligand-bound GR conformation, and thereby recruitment of co-modulators (Caratti et al., 2015). However, GR can also affect other cellular processes through a non-transcriptional pathway, for example mitotic spindle function (Matthews et al., 2011). One major and consistent effect of GC treatment is loss of tissue integrity, and impaired wound healing. In part, this results from reduced epithelial, macrophage and fibroblast migration (McDougall et al., 2006; Hardman et al., 2005). This programme has not received much attention, but may serve as a model to understand the distinct actions of GCs. Therefore, we used an epithelial cell model to measure migratory responses to GC.

Our initial studies sought to mathematically model the walk properties of cells under basal conditions, to provide a solid baseline for GC comparison. Our cell walk characteristics fitted an α-stable distribution, and the impact of GC altered the parameters in such a way that longer steps were selectively reduced in favour of shorter steps, thereby impairing the searching behaviour of cells. This real-time, individual cell tracking permitted the kinetics of response to be measured, and here the surprising finding was the very rapid onset of action with significant deviation from control cells within 40 min of treatment. This rapid onset of action was similarly seen with the GR antagonist RU486 and with further non-steroidal ligands. That RU486 failed to oppose the agonist effects raised a question of specificity, which was addressed in siRNA studies in epithelial cells, and by replicating the migration assay in macrophages, which permitted genetic loss of GR to be tested. The rapid onset of effect, and paradoxical full agonist phenotype seen with RU486, suggested an unconventional mechanism of action, which was supported by showing that no new mRNA synthesis is required. Although we have previously detected inhibition of Rac1 activity soon after GC treatment in podocytes, in that study we were unable to establish that the Rac1 effect was required for the GC effect, and the time for a Rac1 inhibitor to reduce cell movement was prolonged (>10 h), and also strikingly different to that seen with GC exposure (McCaffrey et al., 2017). For these reasons we did not pursue a role for Rac1 in the current work. Here, we also employed selective GR ligands with well-characterised differences in GR nuclear translocation kinetics in order to gain further insights into mechanism of action. We showed that rapid nuclear translocation did not associate with rapid inhibition of migration, but rather a GR ligand with a markedly slow GR translocation rate was still able to affect cell migration rapidly, even while predominantly residing within the cytoplasm. This is consequently a well-documented model of a truly non-genomic mechanism of GR action.

In pursuit of the mechanism of action for rapid changes in cell motility induced by GR activation, we analysed changes in the cytoskeletal architecture, using real-time imaging of microtubule growth. Microtubule kinetics are inverse to cell migration velocity, with increased microtubule polymerisation making cells less able to change conformation, and to migrate. We selected a 4-h time exposure to analyse microtubule polymerisation, as we had shown that by this time point significant changes in cell migration were seen, and there were no changes in gene transcription that could plausibly affect cell migration and, as well as that, blockade of new gene transcription had no impact on the GC effect. Microtubule polymerisation is driven by increased tubulin acetylation (Hubbert et al., 2002; Boggs et al., 2015; Zhang et al., 2003). Tubulin acetylation is tightly controlled by the opposing actions of the α-tubulin acetyltransferase αTAT1 and the deacetylase HDAC6 (Castro-Castro et al., 2012; Liu et al., 2012). HDAC6 is localised predominantly in the cytoplasm, where it directly interacts with microtubules and catalyses tubulin deacetylation along the length of the microtubule track (Asthana et al., 2013; Miyake et al., 2016). Moreover, HDAC6 has shown to prefer deacetylation of tubulin dimers over polymerized microtubules (Skultetyova et al., 2017).

Here, we show that a non-conventional GR mechanism of action is responsible to the rapid onset of cell migration inhibition. We also see the same mechanism at play at later time points, with RU486 failing to inhibit the migratory phenotype even up to 24 h. It is hard to exclude the involvement of additional mechanisms of action at later time points, as with all studies of GC action, the longer exposures result in a greater number of responses, as secondary and tertiary events come into play. We cannot block gene transcription for long periods of time as this is toxic to the cells, and therefore makes the cellular phenotype hard to interpret, but we can use the RU486 to block transcriptional activity of the GR. In these cells, and under these conditions, RU486 is essentially without any agonist activity. RU486 alone exerts a similar impact on cell migration as that of the full agonists, identifying a non-transcriptional mechanism of action.

The GR rapidly translocated to the nucleus after addition of GC, but despite its mainly nuclear location there is evidence for very rapid cycling on and off recognition sites in DNA, and shuttling between the cytoplasm and nucleus, so providing an explanation for persisting engagement with the HDAC6 enzyme. We observed GR–HDAC6 interaction in our FCCS studies, which showed cytoplasmic interaction 1 h after ligand addition, when bulk GR is mainly nuclear (Paakinaho et al., 2019). Therefore, taken together, we have evidence of an unconventional mechanism of GR action that does not involve target gene transactivation. We do, however, find a novel interaction between the activated GR and HDAC6, and show that the HDAC6 is required to mediate the GC effect.

Our studies identify a previous unrecognised mechanism of GR action with involvement of a protein–protein interaction circuit targeting HDAC6. We were able to show that the HDAC6 dependence of the GC loss of long-step length migration and the rapid cellular response, coupled with a lack of requirement for new gene transcription pointed to a direct mechanism of action with a pathway connecting activated GR and the HDAC6 protein. We were not able to show HDAC6–GR interaction by co-immunoprecipitation, but FCCS studies identified a fraction of the cytoplasmic HDAC6 pool as interacting with GR, with resultant changes in movement kinetics, implying a change in molecular complex formation. The unique cytoplasmic preference for GR amongst the nuclear receptors may explain its capacity to interact with cytoplasmic enzymes, such as HDAC6. Our data support a GR-driven change in HDAC6 behaviour as the mechanism explaining rapid-kinetic loss of cell movement in response to GC exposure.

Defective cell migration in response to GC has widespread consequences including defective tissue repair, and loss of barrier function. Identification of a new mechanism of GC action has implications for attempts to design novel GR ligands, with reduced off-target effects, but also the screening for potent GR ligands capable of engaging this pathway to treat exuberant wound-healing, such as keloid.

The identification of a coherent non-genomic GR mechanism of action leading to a clinically relevant cell migratory phenotype offers new insight into the diversity of GC action. This pathway underlines the difficulty in developing specific anti-inflammatory GR ligands, exemplified by identical action of GR antagonists and agonists on cell migration. In addition, the inhibition of epithelial cell migration is also observed in macrophages, providing a new insight into the anti-inflammatory and immune suppressive functions of GCs, which have largely been focused on chemokine production, adhesion molecule expression, cell survival and enzyme production. Taken together, we elucidate a newly discovered non-genomic pathway of GC action affecting cell migration, with proximal impacts on tissue integrity, repair, and innate immune function.

## MATERIALS AND METHODS

### Mouse models

Colonies of LysM-GR^−/−^, mice were maintained in the University of Manchester Biological Services Facility (BSF) with the Home Office license PPL 70-8768. All genetically modified mice were created on a C57BL/6 background and housed in 12 h:12 h light/dark (L:D) cycles with food and water supplied *ad libitum*. All protocols were approved by the University of Manchester Animal Welfare and Ethical Review Body and the Animals (Scientific Procedures) Act 1986 UK Home Office guidelines were strictly adhered to. Conditional targeted cre positive mice, were sex and age matched with floxed/floxed littermate controls; females aged 10-14 weeks were used for *in vivo* experiments and PECs for *ex vivo* were harvested. Genotyping was performed on all experimental animals.

### Cell culture

Human lung epithelial carcinoma (A549) and human cervical adenocarcinoma (HeLa) cells (ATCC, Teddington, UK) were cultured in high glucose (4500 mg/l) Dulbecco's modified Eagle's medium (DMEM; D6429, Sigma) with L-glutamine, sodium bicarbonate, sodium pyruvate and supplemented with 10% heat-inactivated foetal bovine serum (FBS; F9665, Invitrogen, Paisley, UK) or 10% charcoal-stripped fetal bovine serum (cFBS; #12676029, Invitrogen, Paisley, UK) at 37°C in 5% CO_2_.

### Antibodies and reagents

Antibodies used were: rabbit polyclonal anti-GR (24050-1-AP) used at 1:1000 dilution for western blots, purchased from ProteinTech; monoclonal mouse anti-phospho-Ezrin^Thr567^, radixin^Thr564^ and moesin^Thr558^ (#3141) used at 1:1000 dilution for western blots, monoclonal rabbit phospho-Src^Tyr416^ (#6943) used at 1:1000 dilution for western blots, monoclonal rabbit GAPDH (#2118) used at 1:2500 dilution for western blots, monoclonal rabbit anti-phospho-Akt^Ser473^ (#4060) used at 1:1000 dilution for western blots, and monoclonal rabbit acetyl-α-Tubulin^Lys40^ (#5335) used at 1:1000 dilution for western blots and 1:200 dilution for immunofluorescence, purchased from Cell Signaling Technology; onoclonal mouse anti-α-tubulin (T5168) used at 1:5000 dilution for western blots and 1:500 dilution for immunofluorescence purchased from Sigma; and polyclonal rabbit anti-αTAT1 (HPA046816) used at 1:100 dilution for immunofluorescence, purchased from Atlas Antibodies. Mouse IgG horse radish peroxidase (HRP)-linked whole antibody (NXA931) and rabbit IgG HRP-linked whole antibody (NA934) were purchased from GE Healthcare both used at 1:2500 dilution for western blots.

Plasmids used were N1-HDAC6-eGFP and GRα-GFP (Addgene #47504); the N1-HDAC6-eGFP plasmid was constructed by amplifying the cDNA of human HDAC6 from the plasmid pcDNA3.1(+)-flag-HDAC6 (Addgene #13823) and cloning into the pEGFP-N1 vector (Clontech #6085-1) using a QuikChange site-directed mutagenesis kit (Agilent Technologies,. La Jolla, CA, USA). All constructs were verified through sequencing HaloTag-HDAC6, HaloTag-GR (FHC10483), and pHaloTag vector were purchased from Promega. pBOS-H2B-GFP was purchased from BD Biosciences.

siRNAs used were AllStars Negative Control siRNA (SI03650318), GR siRNA (SI02654764), and αTAT1 siRNA (S104145162) purchased from Qiagen.

Reagents used for cell treatments were Rhodamine–phalloidin (R415), purchased from Invitrogen; dexamethasone (dex, D4902), mifepristone (RU486, M8046), nicotinamide (N3376), tubacin (SML0065), TSA (T8552), fluticasone propionate (FP, F9428), Hoechst 33342 (#14533) and DMSO (D2650) purchased from Sigma; ITSA1 (CAS 200626-61-5) purchased from Santa Cruz Biotechnology and HaloTag TMR Direct ligand (G2991) was purchased from Promega. GRT7 and GW870086X were developed by GlaxoSmithKline. Unique materials used are available from the authors or from standard commercial sources outlined above.

### Chemotaxis migration assay

The chemotaxis migration assay was performed in 24-well Millicell hanging cell culture inserts (Millipore, MCEP24H48) with an 8 µm polyethylene terephthalate membrane pore. A549 cells were pre-conditioned to 100 nM dex or vehicle control (DMSO) for 48 h (37°C/5% CO_2_). Cells were suspended in serum-free DMEM and seeded into the upper chamber of the Transwell insert (2.5×10^4^ cells/well). The lower chamber was filled with FBS to act as the chemoattractant. 100 nM dex or vehicle control was added to the upper and lower compartments of the Transwell. The cells are incubated for 24 h (37°C/5% CO_2_) to allow chemotaxis to occur. Following incubation, the cells were fixed in 4% paraformaldehyde (PFA) for 15 min at room temperature. Any cells that did not migrate were removed from the upper side of the membrane with a cotton swab. Cells are stained with Crystal Violet (5 mg/ml in 2% ethanol) for 30 min at room temperature. The inserts were washed twice in distilled H_2_O and excess stain was removed mechanically from the upper side of the membrane. The migrated cells were solubilised in 2% SDS overnight at room temperature and absorbance was read at 560 nm using a Glomax plate reader (Promega). Chemotaxis was quantified as a percentage relative to that in the vehicle control.

### Cell stopper migration assay

Migration assay was performed using an Oris 96-well plate with Oris Cell Seeding Stoppers (Platypus Technologies, CMA1.101) according to the manufacturer's instructions. A549 cells were seeded in DMEM plus 10% FBS (10^5^ cells/well) into an Oris 96-well plate containing Oris Cell Seeding Stoppers and incubated for 18 h (37°C/5% CO_2_) to allow attachment. Following incubation, stoppers were removed and cells washed with 1× PBS. The medium was replaced with DMEM plus 10% cFBS, and cells were treated with 100 nM dex or vehicle control (DMSO). The cells were incubated for 48 h to allow migration into the detection zone to occur (37°C/5% CO_2_). Reference wells had Oris Cell Seeding Stoppers left in place to act as the no migration controls. Cells were washed with 1× PBS to remove any debris/unattached cells, fixed in 4% PFA for 40 min at 4°C, and stained with Hoechst 33342 (2 µg/ml; #14533; Sigma) for 5 min at room temperature to label DNA. Images were collected on an Axio Observer A1 (Axiovision) inverted microscope using a 2.5×/0.07 NA Plan-Apochromat objective and captured using a Axio Cam HRc (Axiovision) through MetaVue Software (Molecular Devices). Specific band pass filter sets for DAPI were used to prevent bleed through from one channel to the next. Images were processed and quantification of migration was achieved using ImageJ (http://rsb.info.nih.gov/ij) (Schneider et al., 2012). Images were thresholded for high intensities, converted into binary format, then analysed as particles to determine the area of the detection zone covered with cells, using the no migration controls for reference. Migration was then quantified as the percentage of the detection zone covered with cells, relative to that of the vehicle control.

### Scratch wound healing assay

A549 cells were seeded in DMEM plus 10% cFBS (2×10^4^ cells/well) into a 96-well ImageLock plate (Essen Bioscience, #4379) and allowed to adhere for 24 h (37°C/5% CO_2_). Simultaneous, uniform scratch wounds were induced in each well with the WoundMaker tool (Essen Bioscience) and wells were washed twice in DMEM plus 10% cFBS to remove debris. Cell migration into the wound was acquired immediately following administration of a dose response of dex, RU486 and hydrocortisone (0.1 nM, 1 nM, 10 nM, 100 nM, 1 µM and 10 µM) along with the vehicle control (DMSO). Images were taken at 30 min intervals over 24 h (37°C/5% CO_2_) on an Incucyte Zoom Live-Cell Analysis system using a 10×/0.3 NA Plan Fluor OFN25 (DIC L/N1) objective in brightfield. Cell migration was analysed and quantified using Incucyte Zoom software.

### Live-cell brightfield migration

A549 cells were seeded in DMEM plus 10% cFBS (5×10^4^ cells/well) into a glass-bottomed 24-well plate (Greiner, #82050-898) and allowed to adhere for 24 h (37°C and 5% CO_2_). Cell migration was monitored following treatment with 100 nM dex, 100 nM RU486, 100 nM tubacin, 100 nM 086X, 3 nM GRT7 or vehicle control (DMSO) in real-time (37°C/5% CO_2_). Images were captured over 24 h at intervals of 10 min on a Leica TCS SP5 AOBS inverted confocal using a 20×/0.5 NA Plan Fuotar objective in brightfield. Cells were tracked using a wavelet plugin on IMARIS Pro Plus software (MediaCybernetics) developed by Dr Egor Zindy (University of Manchester, UK).

### Transfection

Transfections were performed with Fugene 6 reagent (E2691; Promega) used at a ratio of 3:1 volume-to-weight ratio with DNA. Fugene 6 was pre-mixed with RPMI medium (serum-free) for 5 min prior to incubation with DNA for 15 min at room temperature. Transfections were performed over 24 h at 37°C/5% CO_2_. Small interfering RNA (siRNA) transfections were performed with Lipofectamine RNAiMAX reagent (#13778150; ThermoFisher Scientific) as described in the manufacturer's instructions and performed over 48 h at 37°C/5% CO_2_.

### Live-cell immunofluorescence migration

A549 cells were seeded onto glass-bottomed 24-well plates (#662892; Greiner) at 25,000 cells per well in DMEM plus 10% charcoal-stripped FBS and left to adhere overnight. Cells were transiently transfected with 0.5 µg pBOS-H2B-GFP and left to incubate for 24 h at 37°C/5% CO_2_. Cells were treated with vehicle (DMSO) or dex (100 nM) and live-cell imaging was performed for 48 h using a Nikon TE2000 PFS microscope. Images were acquired every 5 min using a 20× Plan Apo objective and the Sedat filter set (Chroma 89000). Cells were maintained at 37°C/5% CO_2_ throughout imaging. The images were collected using a Coolsnap HQ (Photometrics, USA) camera and raw images were processed using ImageJ.

### RT^2^ profiler-PCR array

A human cell motility RT^2^ Profiler PCR array (384-well plate) was used to assay gene expression changes following treatment (PAHS-128Z; Qiagen). Cells were treated as required, then lysed and RNA extracted using an RNeasy kit including the on-column DNase digestion step (#74104; Qiagen). 400 ng RNA was reverse transcribed (#330401; Qiagen) and cDNA samples were added to the reaction plate and real-time PCR acquired using an ABI qPCR machine (Applied Biosystems, CA, USA). Cycle threshold (C_T_) values were exported and analysed according to the manufacturer's instructions by RT^2^ profiler PCR array data analysis software (http://dataanalysis.qiagen.com/pcr/arrayanalysis.php; Qiagen). Five housekeeping genes were assessed and the most suitable, RPLP0, was selected for normalisation of gene expression. A complete data set is provided in Table S1, presented as a fold change over the vehicle control and 95% confidence interval for each gene analysed.

### Quantitative RT-PCR

A549 cells were treated as required, then lysed and RNA extracted using an RNeasy kit including the on-column DNase digestion step to remove genomic DNA (#74104; Qiagen). 1 µg of RNA was reverse transcribed (#4387406; Applied Biosystems) and analysed by qPCR using Sybr Green detection. Gene expression was assessed using the following primer pairs: glyceraldehyde-3-phosphate dehydrogenase (GAPDH), forward primer, 5′-GAAGGTGAAGGTCGGAGT-3′, reverse primer, 5′-CATGGGTGGAATCATATTGGAA-3′; TSC22D3, forward primer, 5′-TGTGGATGAGGGATGAACAA-3′, reverse primer, 5′-ACCCGCTACAGACAAGCTTT-3′; FK506 binding protein 5 (FKBP5), forward primer, 5′-TGTCTCCCACGTGTGTATTAT-3′, reverse primer, 5′-TTTGCTCAGAACCACTCACAC-3′; pyruvate dehydrogenase kinase 4 (PDK4), forward primer, 5′-CGGGATCAAAGTGGGTCTAC-3′, reverse primer, 5′-GGAGGAAACAAGGGTTCACA-3′. Data were analysed by the ΔΔC_T_ method and normalised to GAPDH.

### Immunoblot analysis

Cells were treated as described in the results and lysed on ice for 30 min with modified RIPA buffer (50 mM Tris-HCl pH 7.4, 1% NP-40, 0.25% sodium deoxycholate, 150 mM NaCl, 1 mM EDTA) supplemented with protease inhibitor (#04693124001, Roche) and phosphatase inhibitor cocktails (P5726; P0044; Sigma). Cell lysates were scraped into 1.5 ml Eppendorf tubes, cleared by centrifugation at 14,000 ***g*** for 10 min at 4°C. Supernatants were collected and protein concentration determined by Bradford assay #23236; Thermo Fisher Scientific. Lysates were resuspended to 1 mg/mL in 1× Laemmli buffer (0.125 M Tris-HCl pH 6.8, 0.1% SDS, 20% glycerol, 0.2% β-mercaptoethanol and 0.001% Bromophenol Blue) and boiled at 95°C for 10 min. Lysates were electrophoresed on Tris-glycine (4-20%) Mini-PROTEAN TGX precast polyacrylamide protein gels (15-well, 15 µl per lane) (#4561096, BioRad, Hertfordshire, UK) using 1× Tris-glycine running buffer (#1610732; BioRad) and run at 130 V for 60 min at room temperature. Gels were transferred onto nitrocellulose membranes using transfer buffer (192 mM glycine, 25 mM Tris base, 20% methanol) and run at 90 V for 60 min at 4°C. Membranes were blocked in blocking buffer (2% skimmed milk, 150 µM NaCl and 0.1% Tween-20) for 2 h at room temperature and incubated with relevant primary antibodies diluted in blocking buffer overnight at 4°C. Membranes were washed 3× in wash buffer (0.3% milk, 48 mM Tris-HCl pH 7.6, 24.8 mM Tris base, 0.1% Tween-20) for 10 min and incubated with relevant horseradish peroxidase (HRP)-tagged secondary antibodies diluted (1:5000) in wash buffer for 1 h at room temperature. Membranes were washed three times in wash buffer and exposed to enhanced chemiluminescence (ECL) Clarity reagent (#1705060; BioRad) for 2 min. Protein bands were visualised using BioMax MR photographic film (#V8572786; GE Healthcare).

### HaloTag pulldown assay

A549 cells were seeded onto 15 cm^2^ dishes at 300,000 cells per ml in CSM and left to incubate for 24 h at 37°C/5% CO_2_. Cells were transiently transfected with either HaloTag-GR (10 µg) or HaloTag-HDAC6 (10 µg) and left to incubate for 24 h. Cells were treated with vehicle (DMSO) or dex (100 nM) for 1 h and washed twice with ice-cold 1× PBS. Cells were gently scraped into conical tubes and centrifuged at 2000 ***g*** for 10 min at 4°C. Supernatant was discarded and cell pellets were stored overnight at −80°C prior to lysis. Before cell lysis, HaloTag resin (G1912; Promega) was mixed to obtain uniform suspension and 200 µl resin was dispensed into 1.5 mL Eppendorf tubes per treatment condition. Tubes were centrifuged at 800 ***g*** for 1 min, supernatant discarded, and resuspended in 800 µl resin equilibration buffer (100 mM Tris-HCl, 150 mM NaCl, 0.005% IGEPAL CA-630). Tubes were centrifuged at 800 ***g*** for 2 min and supernatant discarded. Resin was washed an additional 3× in equilibration buffer. Cell pellets were thawed on ice and resuspended in 300 µl lysis buffer (50 mM Tris-HCl pH 7.5, 150 mM NaCl, 1% Triton X-100, 0.1% sodium deoxycholate) supplemented with 6 µl of 50× protease inhibitor cocktail (800 µg/ml benzamidine HCl, 500 µg/ml phenanthroline, 500 µg/ml aprotinin, 500 µg/ml leupeptin, 500 µg/ml pepstatin A and 50 mM PMSF). Cells were passed five times through a 25G needle to complete lysis and centrifuged at 14,000 ***g*** for 5 min at 4°C. Cleared lysates (300 µl) were transferred to new 1.5 ml tubes and diluted in 700 µl 1× TBS (100 mM Tris-HCl pH 7.5 and 150 mM NaCl). 1 ml diluted lysates were mixed with the washed HaloTag resin and left to incubate overnight on a tube rotator at 4°C. Tubes were centrifuged at 800 ***g*** for 2 min and supernatant discarded. Pellets were washed four times in resin equilibration buffer. After the last wash, resin was resuspended.

### Luciferase reporter gene assay

HeLa cells were seeded onto 10 cm^2^ dishes at 50,000 cells per well in CSM and left to adhere overnight. Cells were transiently transfected with luciferase tagged-mouse mammary tumour virus (MMTV-Luc; 2 µg) or luciferase tagged-nuclear factor-κB response element (NRE-Luc; 2 µg) using Fugene 6 reagent (3:1 volume-to-weight ratio with DNA) for 24 h. Cells were re-seeded onto 24-well plates at 50,000 cells per well in CSM and left to adhere overnight at 37°C/5% CO_2_. Cells were treated as specified in the results, and 18 h later each well was washed twice with 1× PBS. 100 µl of Bright Glo lysis buffer (Promega, E2620) was added to each well and left to lyse on ice for 30 min. Cell lysates were transferred to a white, flat-bottomed 96-well plate and luciferase absorbance was read using a luminometer (Glomax, Promega). Ten 1-s reads were taken from each well and the average relative light units (RLUs) determined. Background wells were included that only contained lysis buffer. IC_50_ and EC_50_ values were extrapolated from the resulting dose–response curves using non-linear regression analysis in GraphPad Prism software, with the following equation: Y=Bottom+{Top-Bottom)/[1+10^(LogIC_50_-X)×HillSlope]}. Where X, log of dose or concentration; Y, response; Top and Bottom, plateaus; LogIC_50_ interchangeable with LogEC_50_; HillSlope, slope factor or Hill slope, unitless.

### Fixed-cell immunofluorescence imaging

A549 cells were seeded in DMEM plus 10% cFBS (5×10^4^ cells per coverslip) and allowed to adhere for 24 h (37°C/5% CO_2_). Cells were treated with vehicle or dex (100 nM) for 48 h. Cells were fixed with 4% PFA for 40 min at 4°C and blocked (0.1% Triton X-100, 1% FBS in PBS) for 1 h at room temperature. The remaining incubations were performed at room temperature unless stated otherwise. Coverslips were incubated with primary antibody diluted in blocking buffer overnight at 4°C. After three 5 min washes in PBS, coverslips were incubated with flourophore-conjugated secondary antibody for 2 h. Cells were subsequently stained with rhodamine-phalloidin in PBS (2 µg/ml) for 15 min and then Hoechst 33342 in PBS (2 µg/ml) for 5 min. Following four 5 min washes in PBS, coverslips were mounted using Vectamount AQ (Vector Labs, H-5501). Images were acquired on a Delta Vision RT (Applied Precision, GE Healthcare) restoration microscope using either a 40×/0.85 NA Uplan Apo objective or a 60×/1.42 NA Plan Apo N objective and the Sedat Quad filter set (Chroma 86000v2, VT, USA). The images were collected using a Coolsnap HQ (Photometrics, AZ, USA) camera with a *Z* optical spacing of 0.5 μm. Raw images were then deconvolved using the Softworx software (GE Healthcare) and maximum intensity projections of these deconvolved images processed using ImageJ.

### Live-cell imaging

#### GR trafficking

A549 cells were seeded in DMEM containing cFBS (2.5×10^4^cells/well) into a glass-bottomed 24-well plate (Greiner, #82050-898) and allowed to adhere for 24 h at 37°C/5% CO_2_. Each well was co-transfected (Fugene 6) with 0.25 µg HaloTag-GR (Promega, FHC10483) and 0.25 µg pBOS-H2B-GFP (BD Biosciences, Oxford, UK) and, 6 h later, incubated for 16 h with 0.25 µl Halo ligand (HaloTag TMRDirect, G2991, Promega) to enable HaloTag visualisation. Alternately, wells were transfected with 0.5 µg HaloTag-HDAC6 (Promega, Southampton, UK) and incubated for 16 h with 0.25 µl HaloTag TMR Direct ligand (Promega, G2991). Sub-cellular GR/HDAC6 trafficking was tracked in real-time at 37°C and 5% CO_2_. Images were acquired on a Nikon TE2000 PFS microscope using a 20× Plan Apo objective and the Sedat filter set (Chroma 89000). The images were collected using a Coolsnap HQ (Photometrics, USA) camera and raw images were processed using ImageJ.

#### Fluorescence cross-correlation spectroscopy

A549 cells were seeded onto glass bottomed 35 mm dishes (#627965; Greiner) at 25,000 cells per well in CSM and left to adhere overnight. Cells were transiently co-transfected with HaloTag-GR (500 ng) and HDAC6-eGFP (500 ng) using Fugene 6 reagent (3:1 v/w ratio with DNA) for 24 h. At 6 h post-transfection, cells were treated with 100 nM HaloTMR Direct ligand (G2991; Promega) overnight to visualise HaloTag-GR. The following morning, cells were washed once with CSM before being treated with GC. FCCS was performed using either a Zeiss LSM780 or LSM880 equipped with GaAsP detectors using a plan-apochromat 63×/1.4 NA oil objective. EGFP was excited with 488 nm (0.3%) laser light and emission collected between 500 and 530 nm. Rhodamine–mCherry was excited with 561 nm (0.3%) laser light and emission collected between 580 and 630 nm. Single-point 5×5 s runs FCCS measurements were taken within the cytoplasm and nucleus of individual cells. Zen 2.3 software was used for data collection and correlation-curve fitting. A two-component 3D diffusion model using a fixed structural parameter (S=4) with or without triplet states were fitted for autocorrelation and cross-correlation curves, respectively. FCCS measurements were rejected if counts per molecule (CPM) were <1 kHz, or if they exhibited >10% photobleaching in either the green or red channels. Structural parameter values of 4 and an effective confocal volume size of 0.57 fL were previously measured allowing for determination of molar concentrations (Schneider et al., 2012). The ‘fit’ and ‘confit’ functions in MATLAB 2018a were used to determine *in vivo* dissociation constants and confidence intervals according to the equation (Costes et al., 2004):
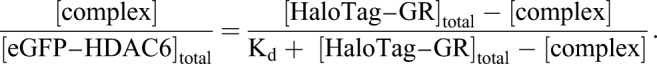


Data points were excluded from the fit if total red or total green concentrations were more than three standard deviations away from mean values. Confidence intervals (0.95, 0.99, etc.) were compared to test for statistical significance. FCCS data represent quantification of three independent experiments from >30 cells. Statistical significance of relative cross-correlation (RCC) between HDAC6 and GR in vehicle- and dex-treated A549 cells (within the cytoplasm and nucleus) was determined using one-way ANOVA followed by Sidak's multiple comparison testing (*P*<0.05).

### Live-cell microtubule dynamics

Microtubule dynamics were monitored in A549 cells transiently transfected with 0.25 µg of EB3-GFP and 0.75 µg pcDNA3 using Fugene 6 reagent and treating with vehicle or dex (100 nM) for 4 h prior to imaging. Cells were selected at random based on the expression of EB3–GFP and images were captured every 0.5 s for 1 min on a Nikon TE2000 PFS microscope using an apo-TIRF 100×/1.49 NA oil objective. The images were collected using a Cascade II EMCCD camera (Photometrics) with a *Z* optical spacing of 0.2 μm. Raw images were then processed using Image J (Schneider et al., 2012).

### Image analysis

For live-cell pBOS-H2B-GFP tracking, A549 cell movement was tracked using the ImageJ plugin Mosaic based on pBOS-H2B-GFP expression. Tracking was performed following the manufacturer's instructions. Co-ordinates of the tracks and the corresponding movies including tracks were exported, from which step length, the distance moved between each image acquisition, and total displacement, the overall distance each cell moved where calculated. For step length, the median step length for each cell was calculated over the duration of tracking and then displayed graphically or a frequency distribution curve of all step lengths from every cell was generated.

Rose-plots were created from 20 cells chosen at random to be displayed as a rose plot. Co-ordinates were transformed so every cell track originated from the *XY* co-ordinates (0,0). Rose plots were generated in GraphPad Prism.

Co-localisation of HDAC6 with actin or tubulin was determined by using ImarisColoc software (Bitplane), which utilises the algorithms developed by Costes et al. (2004) to automate co-localisation.

### Mathematical analysis

Frequency distributions of step lengths were parameterised and fitted to alpha stable distribution using the STBL: Alpha stable distributions functions package for MATLAB (available at https://www.mathworks.com/matlabcentral/fileexchange/37514-stbl-alpha-stable-distributions-for-matlab). The parameters estimated were for media step length (µm), stability exponent (α), skewness (β), scale (γ) and location (δ). A standard resampling strategy was used to validate the parameters. Random sampling with replacement from the original data sets was performed to generate 100 subsets of 15,000 values. These subsets were then parameterised according to an α-stable distribution to derive robust estimates of the standard deviations. The functions package was used to calculate probability distribution functions (PDFs) of the empirical data and from the determined parameters, and plot them on the same axis.

### Live-cell brightfield tracking

A549 cell movement was tracked using Imaris 8.0 software (Media Cybernetics, ltd). Migration was tracked using an autoregressive motion algorithm from cells filtered by size (25 µm) and from tracks filtered by minimum movement speed (above 2.5 µm/minute) to discount stationary debris. Cell migration was depicted as step length between each time point, which was determined using Pythagoras (square root of a^2^+b^2^=c, where a is the position along the *x*-axis, b is the position along the *y*- axis, and c is the step length).

### Analysis of EB3 tracking

A MATLAB-based software package, plusTipTracker (https://omictools.com/plustiptracker-tool) (Applegate et al., 2011; Matov et al., 2010), was used to determine microtubule dynamics (growth, shrinkage and pausing events) from EB3 time-lapse movies. All movies were analysed with the following parameter values, which were determined prior to analysis using the plusTipParamSweepGUI tool within plusTipTracker: maximum gap length, 10 frames; minimum track length, 3 frames; search radius range, 5–10 pixels; maximum forward angle, 20°; maximum backward angle, 10°; maximum shrinkage factor, 1; fluctuation radius, 1.5 pixel. The plusTipGetTracks tool was used to detect and track fluorescently labelled MT plus-end-binding proteins (+TIPs), with only MT growth events being detected in both vehicle and dex treated cells. Overlay images showing the tracks for MT growth speed were generated with the plusTipSeeTracks tool. Raw data was collected from the gs_fs_bs_gl_fl_bl_gd_fd_bd.txt generated by the plusTipGetTracks tool and combined; the frequency of comet growth speeds were determined. Data was subdivided into slow, medium, fast and very fast comets and histograms were generated in GraphPad Prism.

### Statistical analysis of cell movement data

Cumulative distance data for varying-length trajectories (∼1000 cells in each experiment, measurements every 10 min up to 24 h) for cells treated with GW870086X, dexamethasone, GRT7, and RU486 were used to perform statistical test on the median reduction in cumulative distance travelled against corresponding vehicle-treated cells.

For any pair, for example dexamethasone versus vehicle, and at each time point, two distributions of cumulative distances are compared using a non-parametric rank-sum test (using MATLAB), reporting significance (at α=0.0001) of median reduction under treatment (e.g. dexamethasone) with respect to vehicle against the null hypothesis that medians are equal in both distributions.

## Supplementary Material

Supplementary information

Reviewer comments
