# Supplementary material for: Glucocorticoids rapidly inhibit cell migration through a novel, non-transcriptional HDAC6 pathway
Source: J Cell Sci. 2020 Jun 11;133(11):jcs242842. doi: 10.1242/jcs.242842 (PMC7295589; doi:10.1242/jcs.242842)
Supplement: Supplementary information [file joces-133-242842-s1.pdf]

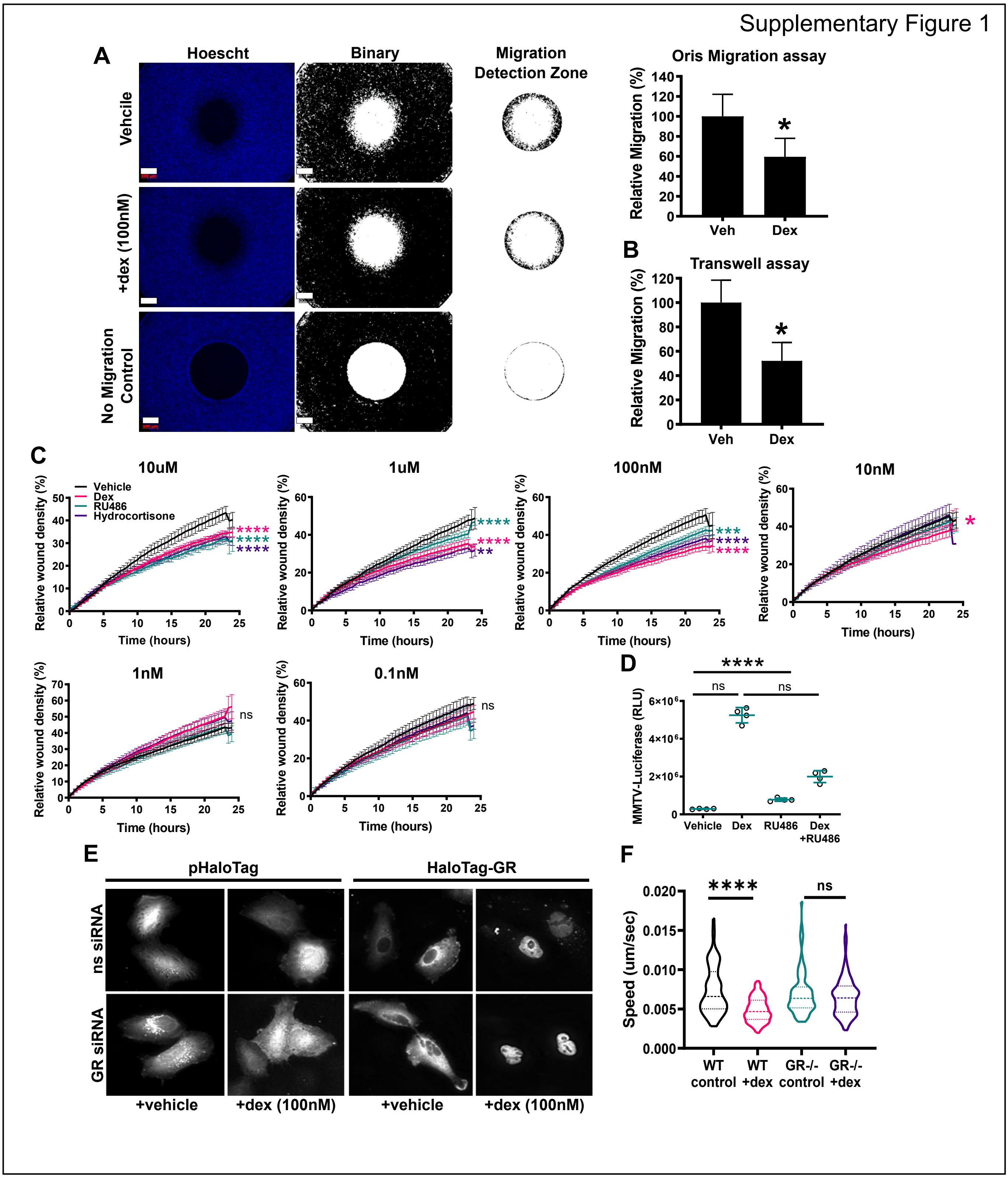

Journal of Cell Science • Supplementary information

Figure S1: Glucocorticoids impair migration at a population level and reduce the motility of individual cells

(A) Representative images and quantification of A549 cell migration in the Oris migration assay following a 48 hour vehicle and Dex (100nM) treatment. Left panel shows nuclear (Hoechst) staining of A549 cells after 48 hours of treatment and a no migration control. Raw images were then thresholded and converted to binary (central panel), and the area of the migration detection zone covered in cells (right panel) was then quantified in ImageJ. Migration is quantified relative to the vehicle control (%), using the no migration control as reference, and represents 4 independent experiments (mean ± SD; *p<0.05, independent t-test). Scale bar represents 500μm.

(B) Relative migration (%) of A549 cells using transwell chemotaxis migration assay in response to 48 hours vehicle or dex (100nM) treatment. Graphs represent quantification of 3 independent experiments (mean ± SD; *p<0.05, independent t-test).

(C) Relative wound density (%) of A549 cells in response to 24 hours vehicle, dex, RU486, and hydrocortisone dose response (0.1nM, 1nM, 10nM, 100nM, 1μM, and 10μM) in a scratch wound healing assay. Graphs represent quantification of at least 2 independent experiments (mean ± SEM, *p<0.05, one-way ANOVA).

(D) Transcriptional activation of the MMTV-luciferase promoter, which was transiently transfected into HeLa cells, in response to 24 hours vehicle, dex (100nM), RU486 (100nM), and dex+RU486 treatment. Graph represents quantification of 3 independent experiments (mean ± SEM, *p<0.05, one-way ANOVA).

(E) Representative confocal images of HaloTag-GR rescue in GR knockdown cells (using GR siRNA #11) in response to 1 hour vehicle or dex (100nM) treatment. Images are representative of two independent experiments. Confocal images were acquired on a Zeiss LSM780 with Confocor 3 mounted on an AxioObserver Z1 microscope at 40x magnification.

(F) Speed (μm/s) of PECs from WT, and GR-null macrophages in response to 24 hours vehicle or dex (100nM) treatment. Graph represents quantification of 2 independent experiments from >1000 cells (mean ± SD, ****p<0.0001, one-way ANOVA).

Journal of Cell Science • Supplementary information

Supplementary Figure 2

A

	Median step size	α	β	γ	δ
Vehicle	0.575	1.4935±0.01	1	0.2475±0.002	0.8091±0.006
Dex	0.440	1.6537±0.01	1	0.1899±0.001	0.5541±0.003

B **C**

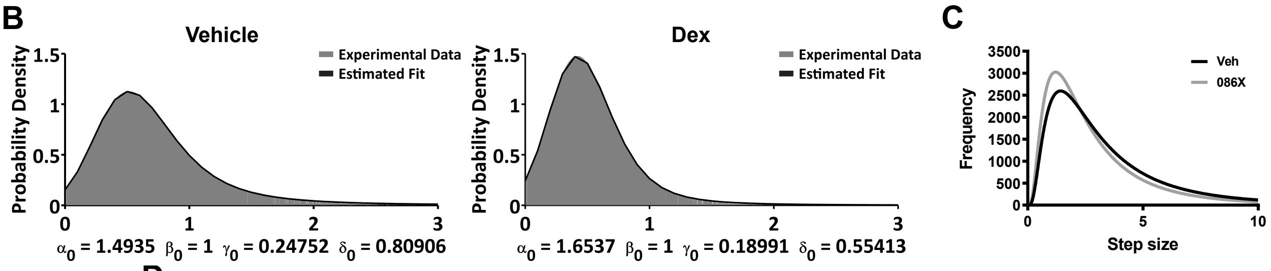

D

	Median step size	α	β	γ	δ
Vehicle	2.484	1.326±0.01	1	1.057±0.008	4.085±0.06
086X	2.116	1.209±0.009	1	0.898±0.007	4.420±0.1

E

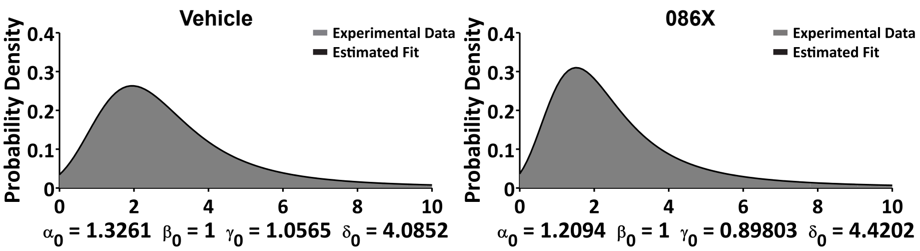

Journal of Cell Science • Supplementary information

Figure S2: Changes in total net displacement following treatment of A549 cells with either conventional steroidal or selective ligands

(A – B) Mathematical modelling of step length data from Fig 1A-C following the full 48 hours of imaging.

(C-E) Represents data from three independent experiments, tracking data points from >1350 cells per treatment condition.

(A) Estimated alpha stable distribution parameters of A549 cell migration in response to 48 hours vehicle and dex (100nM). Parameterisation was performed on step length data in MATLAB, estimating values for media step length (μm), stability exponent (α), skewness (β), scale (γ), and location (δ). Standard deviation estimates were generated by parameterising 100 randomly sampled subsets of the 15,000 values from the original data sets.

(B) Probability density function (PDF) plots of the experimental step length data (dark grey) and PDF plots generated from the estimated alpha stable distribution parameters (black line) in response to 48 hours vehicle and dex (100nM) treatment. Plots were produced in MATLAB. (A-B) Represents data from two independent experiments, consisting of 52 vehicle treated and 54 dex treated cells.

(C) Frequency distribution curves of A549 step lengths (μm) in response to 24 hours of vehicle and 086X (100nM) treatment.

(D) Estimated alpha stable distribution parameters of A549 cell migration in response to 24 hours of vehicle and 086X (100nM) treatment.

(E) Probability density function (PDF) plots of experimental step length data (dark grey) and estimated alpha stable distribution parameters (black line) in response to 24 hours of vehicle and 086X (100nM) treatment.

Journal of Cell Science • Supplementary information

Supplementary Figure 3

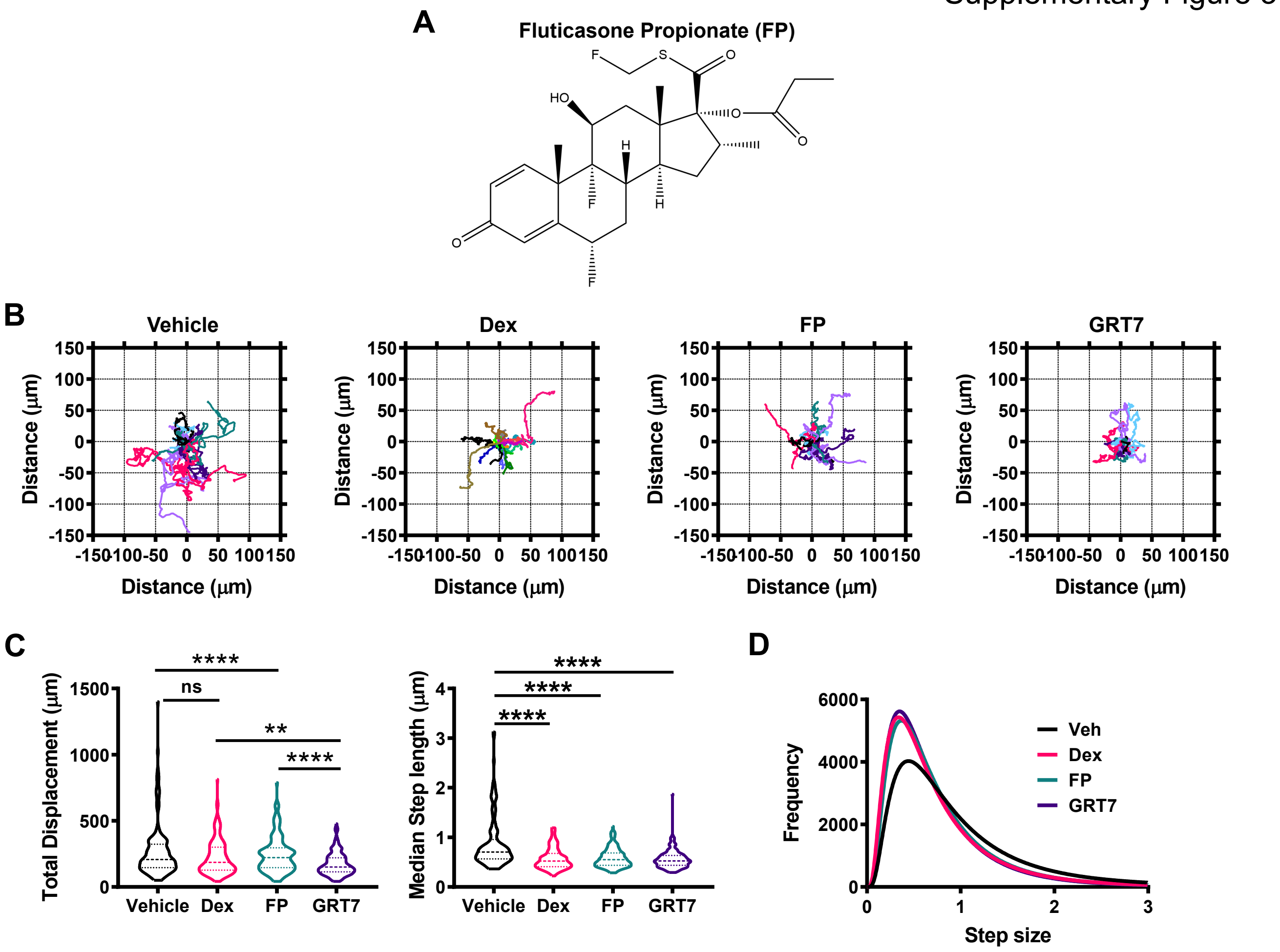

E

	Median step size	α	β	γ	δ
Vehicle	0.705	1.290 ± 0.01	1	0.3005 ± 0.002	1.251 ± 0.02
Dex	0.513	1.493 ± 0.01	1	0.2323 ± 0.002	0.736 ± 0.007
FP	0.537	1.524 ± 0.01	1	0.2348 ± 0.002	0.738 ± 0.006
GRT7	0.504	1.565 ± 0.02	1	0.2233 ± 0.002	0.674 ± 0.006

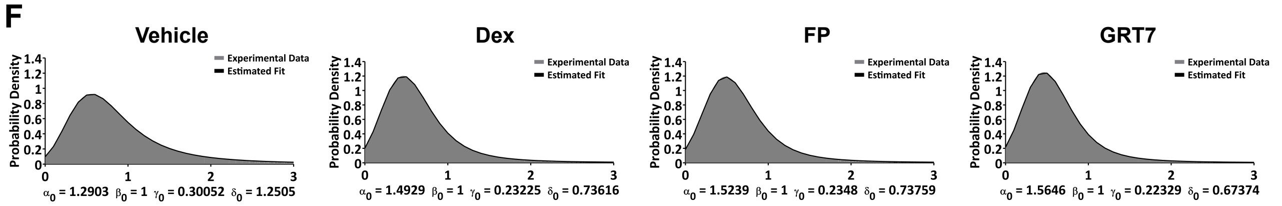

Journal of Cell Science • Supplementary information

Figure S3: Comparison of the effects of steroidal and non-steroidal ligands on cell movement

(A) 3D chemical structure of fluticasone propionate (FP).

(B) Rose plots of A549 cell displacement (µm) in response to 48 hours of vehicle, dex (100nM), FP (3nM), and GRT7 (3nM) treatment. Each coloured line represents the displacement of one cell from its point of origin. Each rose plot is representative of 20 randomly chosen A549 cells.

(C) Violin plots of total displacement (µm) and median step length (µm) of A549 cells in response to 48 hours of vehicle, dex (100nM), FP (3nM), and GRT7 (3nM) treatment. Migration data shown as median ± IQD and represents all cells analysed over two independent experiments (Kruskal-Wallis non-parametric test followed by Dunn's multiple comparisons test; ****p<0.0001, **p=0.002).

(D) Frequency distribution curves of A549 cell step lengths (µm) in response to 48 hours of vehicle, dex (100nM), FP (3nM), and GRT7 (3nM) treatment.

(E) Estimated alpha stable distribution parameters of A549 cell migration in response to 48 hours of vehicle, dex (100nM), FP (3nM), and GRT7 (3nM) treatment. Parameterisation was performed on step length data in MATLAB, estimating values for media step length (µm), stability exponent (α), skewness (β), scale (γ), and location (δ). Standard deviation estimates were generated by parameterising 100 randomly sampled subsets of the 15,000 values from the original data sets.

(F) PDF plots of experimental step length data (dark grey) and estimated alpha stable distribution parameters (black line) in response to 48 hours of vehicle, dex (100nM), FP (3nM), and GRT7 (3nM) treatment. Plots generated in MATLAB

For observed data graphs represent >40,000 data points for each condition for >135 cells.

Journal of Cell Science • Supplementary information

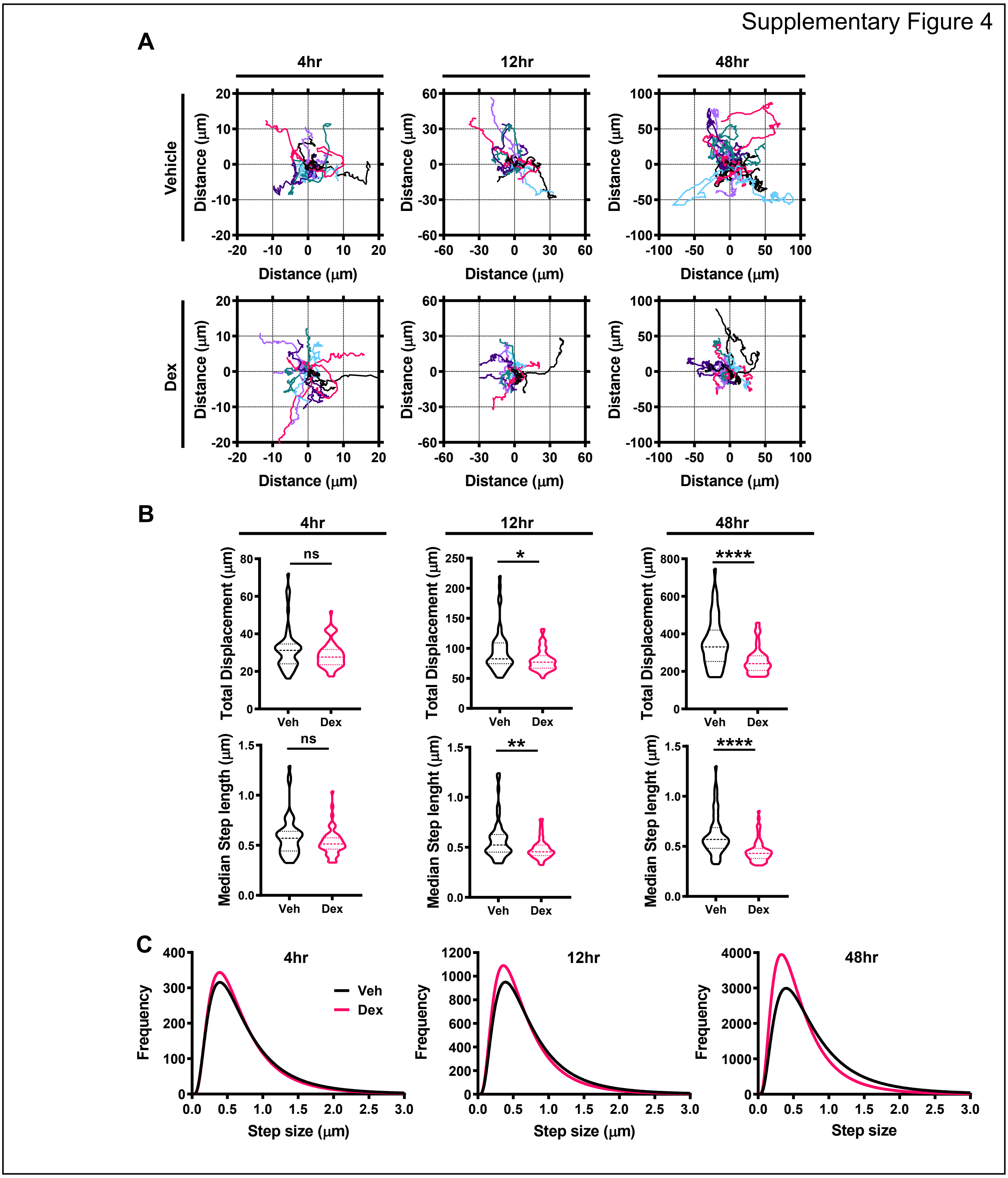

Journal of Cell Science • Supplementary information

Figure S4: Time-course of the dexamethasone effect on cell migration

Cell migration data for cells that were transiently transfected with 0.5µg pBOS-H2B-GFP and incubated for 24 hours at 37°C/5% CO_2 prior to imaging. Cells were treated with vehicle and dexamethasone (dex; 100nM) and images acquired every 5 minutes for 48 hours. Data displayed represents subsampling of migration data from Fig. 1A-C after 4 hours, 12 hours and 48 hours of cell tracking.

(A) Rose plots of A549 cell displacement (µm) in response to 4, 12 and 48 hours of vehicle and dex (100nM) treatment. Each coloured line represents the displacement of one cell from its point of origin. Each rose plot is representative of 20 randomly chosen A549 cells.

(B) Violin plots of total displacement (µm), the overall distance moved by every cell tracked, and median step length (µm), the median distance of the distances moved by a cell between every image acquisition, of A549 cells in response to 4, 12 and 48 hours of vehicle (DMSO) and dex (100nM) treatment. Migration data is representative of 52 individual cells for vehicle treatment and 54 individual cells for dex treatment, across two independent experiments. Each data point represents one individual cell. Median ± IQD (Mann Whitney non-parametric test; ****p<0.0001, **p=0.0083, *p=0.0352).

(C) Frequency distribution curves of all A549 cell step lengths (µm) in response to 4, 12 and 48 hours of vehicle and dex (100nM) treatment.

Journal of Cell Science • Supplementary information

Supplementary Figure 5

A

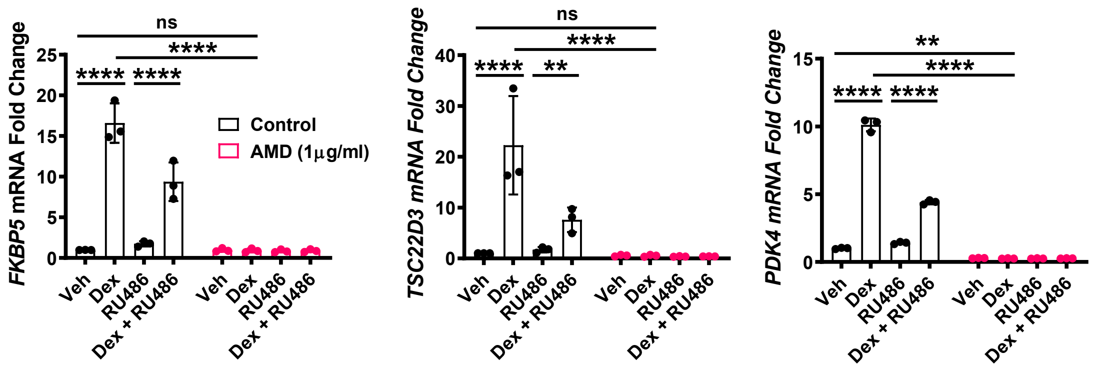

Figure S5: Regulation of GR target genes is dependent on transcription

(A) A549 cells were pre-treated with a vehicle control or actinomycin D (AMD; 1µg/ml) for 1 hour, then treated with either a vehicle control, dex (100nM), RU486 (100nM) or a combination of dex and RU486 for 4 hours. Expression of the GR target genes FKPB5, TSC22D3 and PDK4 was analysed by RT-qPCR. Data is normalised to the housekeeping gene GAPDH and displayed as a fold change over the vehicle control. Graphs (mean ±SD) combine data from at three independent experiments. Statistical analysis was performed by one-way ANOVA followed by Holm-Sidak's multiple comparison (**p<0.005; ****p<0.0001)

Journal of Cell Science • Supplementary information

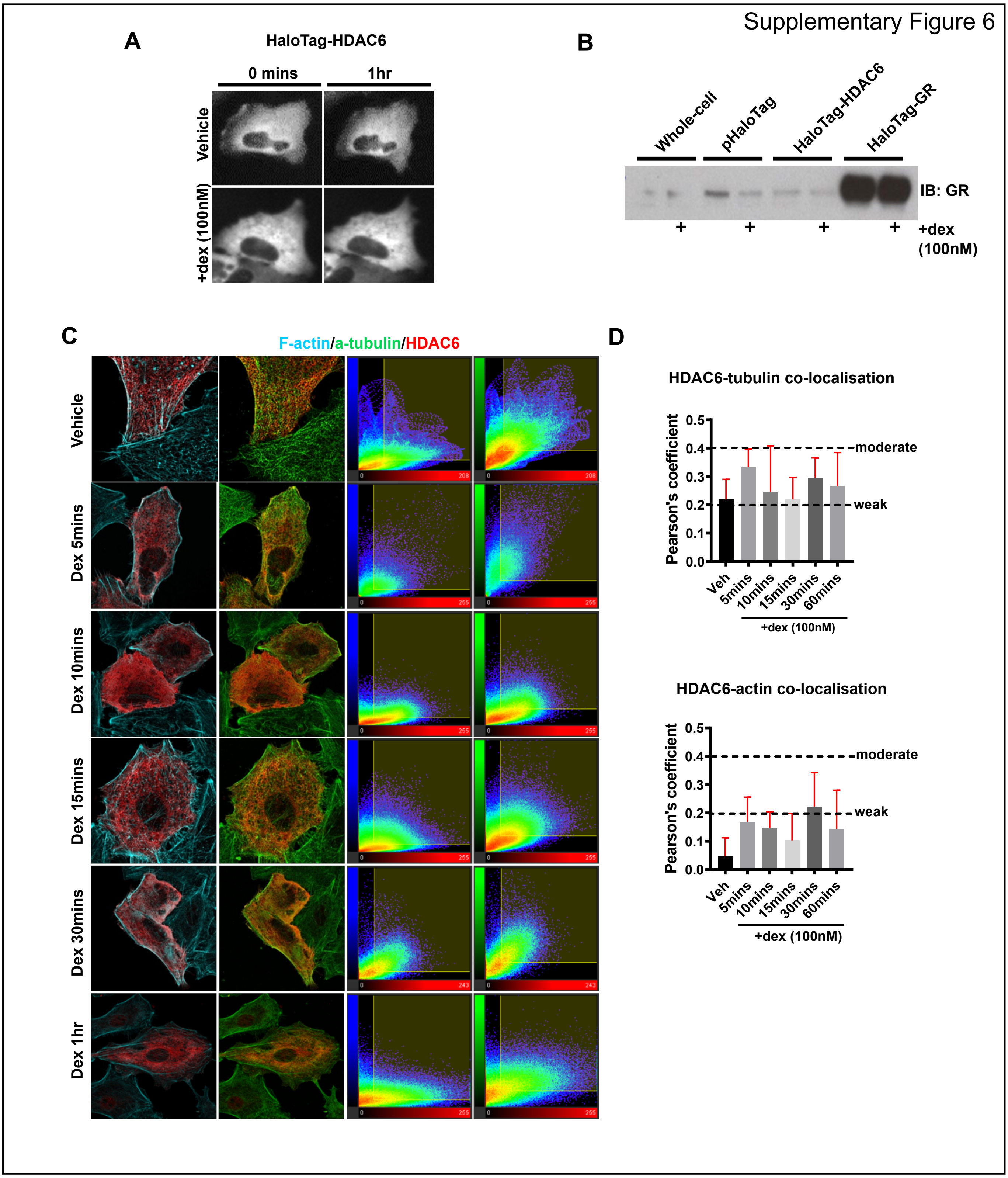

Journal of Cell Science • Supplementary information

Figure S6: HDAC6:-GR interaction and co-localisation with cytoskeletal architecture in response to glucocorticoid

(A) Representative images of HaloTag-HDAC6 tagged labelled with the HaloTMR Direct fluorescent ligand in A549 cells, in response to 1 hour of vehicle or dex (100nM) treatment. Images are representative of two independent experiments. Widefield images were acquired on a Delta Vision RT (Applied Precision, GE Healthcare) restoration microscope at 20X magnification

(B) HaloTag pulldown from A549 cells transiently transfected with HaloTag-GR, HaloTag-HDAC6, or pHaloTag control and immunoblotted for GR and HDAC6 protein expression to demonstrate co-immunoprecipitation in response to 1 hour of dex (100nM) treatment.

(C) Confocal immunofluorescence of HDAC6 (red), F-actin (cyan), and α-tubulin (green) in A549 cells transiently transfected with HaloTag-HDAC6 and co-localisation analysis of HDAC6 v F-actin and HDAC6 v tubulin in response to dex (100nM) time series (5, 10, 15, 30, and 60 minutes).

(D) Pearson's correlation coefficient (PCC) of HDAC6-actin and HDAC6-tubulin co-localisation in response to dex (100nM) time series (0.2-0.4 as weak and >0.4 as moderate co-localisation). PCC was calculated using ImarisColoc software (Bitplane), which utilises the Costes algorithm to automate co-localisation.

Table S1: RT^2 Profiler PCR array data

(A) A549 cells were treated with a vehicle control, dex (100nM), RU486 (100nM) or a combination of dex and RU486 for 4 hours. RNA was extracted and 400ng was reverse transcribed. Expression of 84 genes associated with cell migration was assessed via a human cell motility RT^2 Profiler PCR array. Data is normalised to the housekeeping gene RPLP0 and is displayed as a fold change over the vehicle control, and includes a 95% confidence interval. Fold change values greater than 2 (upregulated) are in red and fold change values less than 0.5 (downregulated) are indicated in blue.

Click here to Download Table S1

Journal of Cell Science • Supplementary information
