# Supplementary material for: Glucocorticoids rapidly inhibit cell migration through a novel, non-transcriptional HDAC6 pathway
Source: J Cell Sci. 2020 Jun 11;133(11):jcs242842. doi: 10.1242/jcs.242842 (PMC7295589; doi:10.1242/jcs.242842)
Supplement: Reviewer comments [file joces-133-242842_review_history.pdf]

Glucocorticoids rapidly inhibit cell migration through a novel, non-transcriptional HDAC6 pathway.

Stephen Kershaw, David J. Morgan, James Boyd, David G. Spiller, Gareth Kitchen, Egor Zindy, Mudassar Iqbal, Magnus Rattray, Christopher M. Sanderson, Andrew Brass, Claus Jorgensen, Tracy Hussell, Laura C. Matthews and David W. Ray
DOI: 10.1242/jcs.242842

Editor: John Heath

Review timeline

Original submission: 13 December 2019
Editorial decision: 19 February 2020
First revision received: 9 April 2020
Accepted: 21 April 2020

Original submission

First decision letter

MS ID#: JOCES/2019/242842

MS TITLE: Glucocorticoids rapidly inhibit cell migration through a novel, non-transcriptional HDAC6 pathway.

AUTHORS: Stephen Kershaw, David Morgan, James Boyd, David Spiller, Gareth Kitchen, Egor Zindy, Mudassar Iqbal, Chris Sanderson, Magnus Rattray, Andrew Brass, Claus Jorgensen, Tracy Hussell, Laura Charlotte Matthews, and David Ray
ARTICLE TYPE: Research Article

We have now reached a decision on the above manuscript. We only have one report at this point but to save more delay I have read the paper myself and agree with the reviewer's remarks.

To see the reviewers' report and a copy of this decision letter, please go to: https://submit-jcs.biologists.org and click on the 'Manuscripts with Decisions' queue in the Author Area. (Corresponding author only has access to reviews.)

As you will see, the reviewer raises a number of criticisms that prevent me from accepting the paper at this stage. They suggest, however, that a revised version might prove acceptable, if you can address their concerns. If you think that you can deal satisfactorily with the criticisms on revision, I would be pleased to see a revised manuscript. We would then return it to the reviewers.

Please ensure that you clearly highlight all changes made in the revised manuscript. Please avoid using 'Tracked changes' in Word files as these are lost in PDF conversion.

I should be grateful if you would also provide a point-by-point response detailing how you have dealt with the points raised by the reviewers in the 'Response to Reviewers' box. Please attend to all of the reviewers' comments. If you do not agree with any of their criticisms or suggestions please explain clearly why this is so.

© 2020. Published by The Company of Biologists under the terms of the Creative Commons Attribution License (https://creativecommons.org/licenses/by/4.0/).

Reviewer 1

Advance summary and potential significance to field

Kershaw et al demonstrate that glucocorticoid receptor agonists and antagonists have similar effects of cell migration, with both producing a reduction in cell motility. The authors attribute this to microtubule stabilisation due to inhibition of HDAC6, resulting in inhibition of motility.
The authors demonstrate an interaction between glucocorticoid receptor and HDAC6 in the cytoplasm using fluorescence cross-correlation spectroscopy (FCCS), as an alternative to Co-IP, which failed to identify GR and HDAC6 in complex, presumable since GR rapidly translocates to the nucleus after agonist or antagonist stimulation.

In general the experiments are well performed with suitable controls. The novelty in this study is provided by the link between GC and altered microtubule dynamics through HDAC6 inhibition. However, there are some issues that should be addressed prior to publication to firm up the conclusions.

Comments for the author

Major comments:

1. A previous report (McCaffrey et al. 2017, doi: 10.1038/s41598-017-06810-y) demonstrated that GCs can reduce Rac1 activity. This effect was observed at the same time point when a reduction in cell motility was observed, suggesting the two go hand in hand. Does dexamethasone have a similar affect on Rac1 activity? Ideally this would be measured in live-cells using the Raichu FRET probes to gain insight into the temporal dynamics of Rac1 activity (since a significant reduction in cell motility is seen <1 hour after addition of dexamethasone). If so, does overexpression of a constitutively active Rac1 construct block the effects of dex on cell motility?

2. It is difficult to determine the time-line of events to justify the conclusions drawn. Whilst microtubule stabilisation might impair cell motility, this could also be a by-product of reduced cell motility (e.g. due to reduced Rac1 activity levels, see comment 1). This is particularly difficult to assess given that the experiments assessing microtubule dynamics (Fig. 4) were conducted after 4 hours of dexamethasone treatment, whereas other experiments were conducted after shorter dex treatment (FCCS, Fig. 6; cytoplasm-to-nucleus translocation of GR. Fig. 3), and an inhibitory effect on migration was observed rapidly after dexamethasone treatment. Presuming that the tight association between GR and HDAC6 (occurring after <1 hour of dexamethasone) is responsible for the changes in microtubule dynamics, then the assessment of microtubule dynamics should be performed after a similar time point, rather than after 4 hours of dexamethasone treatment.

3. Figure 6 appears to be missing statistical analysis (with the exception of panel ‘j’); is there a significant increase in relative cross-correlation of GR bound to HDAC6 in the cytoplasm after dexamethasone?

Minor comments:

1. Figure 1: there seems to be a large difference in cell motility between control groups in the data presented in panels ‘a’ and ‘e’. Is there a reason for this, or just variation between experiments?

2. In the introduction, line 64, the authors write: “GCs interact with the cytoskeleton to facilitate ligand-induced nuclear translocation amongst other functions”. From this text it is not clear precisely what is translocating to the nucleus, please clarify.

© 2020. Published by The Company of Biologists under the terms of the Creative Commons Attribution License (https://creativecommons.org/licenses/by/4.0/).

First revision

Author response to reviewers' comments

Reviewer 1 Comments for the author
Major comments:

1.A previous report (McCaffrey et al. 2017, doi: 10.1038/s41598-017-06810-y) demonstrated that GCs can reduce Rac1 activity. This effect was observed at the same time point when a reduction in cell motility was observed, suggesting the two go hand in hand. Does dexamethasone have a similar affect on Rac1 activity? Ideally this would be measured in live-cells using the Raichu FRET probes to gain insight into the temporal dynamics of Rac1 activity (since a significant reduction in cell motility is seen <1 hour after addition of dexamethasone). If so, does overexpression of a constitutively active Rac1 construct block the effects of dex on cell motility?

We are very familiar with the McCaffrey work. James was my PhD student, and the work was done under my supervision, in my labs. In the McCaffrey work we did see changes in Rac1 in a rather specialised podocyte cell model. However, we were not able to show that the Rac1 changes were important for regulating cell movement. It is for this reason, namely that we just had an association in one rather specific cell model, and were unable to establish a cause-effect relationship, that we moved away from pursuing Rac1 as a possible mechanism explaining the actions of GR on cell movement. We certainly do not want to claim in the current paper that other cell responses to glucocorticoid do not exist, or that engagement with other cytoplasmic signalling molecules does not occur. Here we are able to show the importance of HDAC6, and are able to identify a molecular interaction between the glucocorticoid receptor and HDAC6. Whilst other signalling molecules might be of interest in the future we think that such studies are beyond the scope of the current paper, and in any case our labs are now locked down.
However, we do now cite and discuss the McCaffrey paper in the discussion, as it is relevant.

2.It is difficult to determine the time-line of events to justify the conclusions drawn. Whilst microtubule stabilisation might impair cell motility, this could also be a by-product of reduced cell motility (e.g. due to reduced Rac1 activity levels, see comment 1). This is particularly difficult to assess given that the experiments assessing microtubule dynamics (Fig. 4) were conducted after 4 hours of dexamethasone treatment, whereas other experiments were conducted after shorter dex treatment (FCCS, Fig. 6; cytoplasm-to-nucleus translocation of GR. Fig. 3), and an inhibitory effect on migration was observed rapidly after dexamethasone treatment.

Presuming that the tight association between GR and HDAC6 (occurring after <1 hour of dexamethasone) is responsible for the changes in microtubule dynamics, then the assessment of microtubule dynamics should be performed after a similar time point, rather than after 4 hours of dexamethasone treatment.

We thank the referee for this comment. One of the issues here is the difficulty in performing studies with very short incubation times after which changes in the measured parameters may be small. We think that the causative effect is the modification of the microtubule network which then results in changes to cell movement. This is based on the identification of HDAC6 as a necessary enzyme required to exert the glucocorticoid effect. In addition, we were able to show changes in the microtubule polymerisation. It is true that we did the measurements after a four-hour Dexamethasone incubation, at which time we did not see any gene expression change, and we were also able to show the even a glucocorticoid antagonist was capable of exerting a similar effect.

In our earlier work on Rac1 inhibition we used a Rac1 inhibitor, and compared that against glucocorticoid. The Rac1 inhibitor did not impact cell movement for about 10 hours (Fig7 in the McCaffrey paper). We were also able to show that in fact the Rac1 inhibitor had a different effect compared to that of the glucocorticoid (again in Fig 7, McCaffrey paper). Therefore, although we stand by the Rac1 data in the McCaffrey paper due to time constraints at the time, and the availability of reagents we were not able to take the Rac1 mechanism further. However, we do not think that our previous Rac1 data in any way undermines or contradicts the current GR-HDAC6 mechanism.

© 2020. Published by The Company of Biologists under the terms of the Creative Commons Attribution License (https://creativecommons.org/licenses/by/4.0/).

3.Figure 6 appears to be missing statistical analysis (with the exception of panel ‘j’); is there a significant increase in relative cross-correlation of GR bound to HDAC6 in the cytoplasm after dexamethasone?

We have corrected this and added the result of the test to the figure. There is indeed a significant increase in relative cross correlation between GR and HDAC6 in the cytoplasm after dexamethasone.

Minor comments:

1.Figure 1: there seems to be a large difference in cell motility between control groups in the data presented in panels ‘a’ and ‘e’. Is there a reason for this, or just variation between experiments?

We thank the referee for this comment. As can be seen we have performed a series of analyses using different methodologies through the paper. In all cases we see a reduction in cell movement. The time intervals between sequential measurements do differ depending on the method, and this information is included in the figs and fig legends. Therefore, the absolute numbers relating to cell movement do differ between assays, but the explanation is due to the constraints on frequency of measurement or the timeframe over which cell movement is assessed.

2.In the introduction, line 64, the authors write: “GCs interact with the cytoskeleton to facilitate ligand-induced nuclear translocation amongst other functions”. From this text it is not clear precisely what is translocating to the nucleus, please clarify.

This is an error. We intended to state that the GR interacts with the cytoskeleton. This is well-established, and provides the means for rapid, ATP-dependent nuclear translocation. We have corrected this in the revised manuscript.

<u>Second decision letter</u>

MS ID#: JOCES/2019/242842

MS TITLE: Glucocorticoids rapidly inhibit cell migration through a novel, non-transcriptional HDAC6 pathway.

AUTHORS: Stephen Kershaw, David J Morgan, James Boyd, David G Spiller, Gareth Kitchen, Egor Zindy, Mudassar Iqbal, Magnus Rattray, Chris M Sanderson, Andrew Brass, Claus Jorgensen, Tracy Hussell, Laura C Matthews, and David W Ray
ARTICLE TYPE: Research Article

I am happy to tell you that your manuscript has been accepted for publication in Journal of Cell Science, pending standard ethics checks.

<u>Reviewer 1</u>

Comments for the author

I am satisfied with the changes made by the authors and recommend the paper for publication in JCS.

© 2020. Published by The Company of Biologists under the terms of the Creative Commons Attribution License (https://creativecommons.org/licenses/by/4.0/).
